# Embedding Bifurcations into Pneumatic Artificial Muscle

**DOI:** 10.1002/advs.202304402

**Published:** 2024-04-19

**Authors:** Nozomi Akashi, Yasuo Kuniyoshi, Taketomo Jo, Mitsuhiro Nishida, Ryo Sakurai, Yasumichi Wakao, Kohei Nakajima

**Affiliations:** ^1^ Graduation School of Informatics Kyoto University Yoshida‐honmachi Sakyo‐ku Kyoto 606‐8501 Japan; ^2^ Graduate School of Information Science and Technology The University of Tokyo 7‐3‐1 Hongo Bunkyo‐ku Tokyo 113‐8654 Japan; ^3^ GX Innovation Technology Development Bridgestone Corporation 3‐1‐1 Kyobashi Chuo‐ku Tokyo 104‐8340 Japan

**Keywords:** artificial intelligence, bifurcation theory, control theory, morphological computation, pneumatic artificial muscle, robotics, soft material

## Abstract

Harnessing complex body dynamics has long been a challenge in robotics, particularly when dealing with soft dynamics, which exhibit high complexity in interacting with the environment. Recent studies indicate that these dynamics can be used as a computational resource, exemplified by the McKibben pneumatic artificial muscle, a common soft actuator. This study demonstrates that bifurcations, including periodic and chaotic dynamics, can be embedded into the pneumatic artificial muscle, with the entire bifurcation structure using the framework of physical reservoir computing. These results suggest that dynamics not present in training data can be embedded through bifurcation embedment, implying the capability to incorporate various qualitatively different patterns into pneumatic artificial muscle without the need to design and learn all required patterns explicitly. Thus, this study introduces a novel approach to simplify robotic devices and control training by reducing reliance on external pattern generators and the amount and types of training data needed for control.

## Graphical abstract

This is a study that aims to embed bifurcation structures with periodic and chaotic dynamics into pneumatic artificial muscles.  The reuslts imply that dynamics not present in training data can be embedded through the bifurcation structure. This is expected to lead to a fundamental improvement in the efficiency of learning for bodily control in robotics.

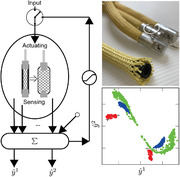

## 1 Introduction

Recent studies have revealed that mechanical devices can be designed to use their body dynamics for desired information processing, for example, in devices like passive dynamic walkers,^[^ ^1^ ^]^ mechanical random number generators,^[^ ^2^ ^]^ and mechanical networks.^[^ ^3^, ^4^ ^]^ Furthermore, the natural dynamics of mechanical bodies not designed for computation can be used as an information processing resource. The complex dynamics observed in soft robotic arms, inspired by the octopus, can be used for real‐time time series processing, including embedding a timer, and controlling the arm through physical reservoir computing (PRC).^[^ ^5^, ^6^, ^7^, ^8^, ^9^ ^]^ Reservoir computing^[^ ^10^, ^11^, ^12^ ^]^ is a recurrent neural network framework characterized by the use of a high‐dimensional neural network with nonlinearity and memory. In PRC,^[^ ^13^ ^]^ the neural network is replaced with physical dynamics. It has been reported that various types of robotic bodies, such as mechanical spring‐mass dampers,^[^ ^14^ ^]^ tensegrity structures,^[^ ^15^, ^16^ ^]^ quadruped robots,^[^ ^17^ ^]^ and fish robots,^[^ ^18^ ^]^ can be exploited as a reservoir. This suggests that body dynamics can be directly exploited for information processing and control without the need for external memory and nonlinearity.

Pneumatic artificial muscles (PAMs), representing soft actuators, have been studied as physical reservoirs. PAMs have been investigated since the inception of soft robotics^[^ ^19^, ^20^, ^21^, ^22^ ^]^ and are central components of various soft machines and devices, including wearable devices^[^ ^23^ ^]^ and robotic arms.^[^ ^24^ ^]^ They offer several advantages such as durability against impact and vibration, a high force‐to‐weight ratio, and low manufacturing costs. Studies exploring PAMs as physical reservoirs have demonstrated their potential. For instance, PAM length sensors can be emulated by other sensory values in the PAM using the PRC framework.^[^ ^25^ ^]^ The air pressure within a rubber tube connected to a PAM, integrated into an assistive walking device, can estimate the posture of the wearer through the use of PRC.^[^ ^26^ ^]^ Moreover, the estimated information can be exploited for the control of assistant timing in the walking device.^[^ ^26^ ^]^ Periodic patterns have been successfully embedded into robotic arms and wearable devices composed of PAMs with PRC closed‐loop control.^[^ ^27^, ^28^ ^]^ Despite the pioneering efforts mentioned above, the computational capabilities of PAMs and the controls they can perform by using these capabilities have not been comprehensively analyzed to date.

The present study provides systematic analyses of the nonlinear and memory capabilities in a PAM as a physical reservoir. It demonstrates the ability to control PAMs in various patterns using these capabilities in real‐world settings. Specifically, the present study demonstrates the embedding of bifurcation structures into the PAM. Bifurcation structures in dynamical systems signify qualitative changes in dynamics, such as periodic and chaotic dynamics, resulting from parameter variations. In central pattern generators, bifurcation structures have been demonstrated to offer capabilities for exploration and self‐organized adaptation in robot control.^[^ ^29^, ^30^, ^31^ ^]^ Recently, it has been reported that artificial neural networks with rich information processing capabilities can reconstruct the entire bifurcation structure by learning a subset of dynamics included in the bifurcation structure.^[^ ^32^, ^33^, ^34^, ^35^, ^36^ ^]^ The present study is the first attempt to realize bifurcation embedment into physical dynamics. Embedding dynamics implies internalizing the central pattern generator into the body, as opposed to external attachment to the robot. In addition, embedding bifurcation structures suggests that it is possible to control various qualitatively different patterns by learning several patterns, without the need to learn all patterns required for robot control. This generalization surpasses traditional machine learning generalizations such as interpolation or extrapolation, as the properties of unseen dynamics in the bifurcation structure are qualitatively distinct from those trained. In concrete terms, the study successfully demonstrates the embedding of chaotic dynamics into a PAM by training solely on periodic dynamics, and vice versa. The present study provides insight into the inherent reduction in the amount and types of data required for learning in robot control.

## 2 Results

### 2.1 Pneumatic Artificial Muscle

**Figure 1:**
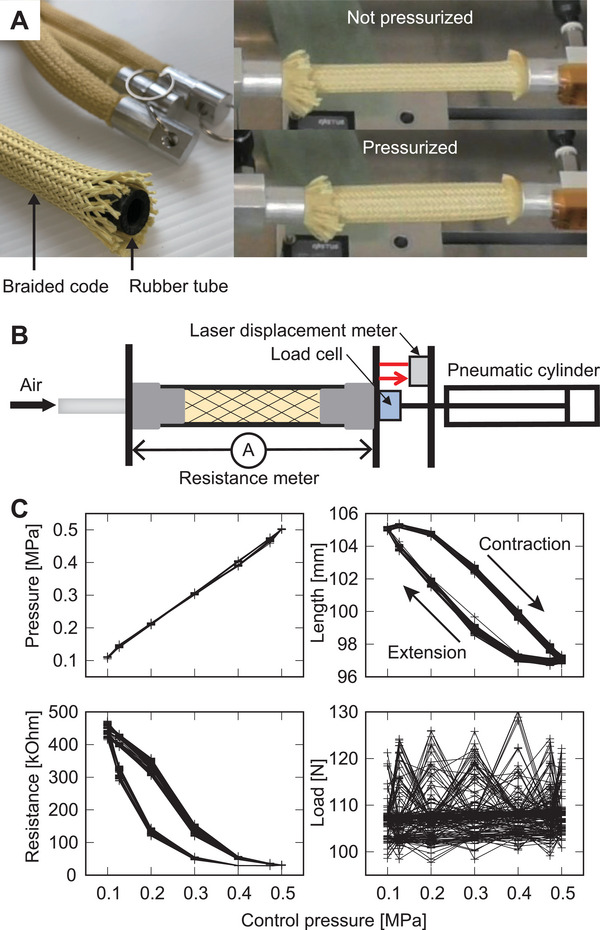
Pneumatic artificial muscles, measurement systems, and pneumatic artificial muscle dynamics. A) No pressurized and pressurized pneumatic artificial muscles. B) Pneumatic artificial muscle measurement systems. C) Sensor responses to a sinusoidal wave input.

The current study used the McKibben PAM (**Figure** **1**A), which consists of a cylindrical rubber tube covered by a braided cord. This PAM is under a nearly constant external load. If the PAM is pressurized, it expands in the radial direction and shrinks lengthwise. We used the PAM as a physical reservoir by injecting an input value as a control pressure and measuring its physical quantities. The measurement system is illustrated in Figure 1B. The inner pressure, length, load, and electric resistance of the rubber were measured. Although the traditional natural rubber has low conductivity and electric resistance value is difficult to measure, the present study increased its conductivity from 1.0 × 10^−3^ to 20 S m^‐1^ by incorporating carbons into the rubber.^[^ ^37^ ^]^ The length of the PAM was 108 mm, with an outer diameter of 11 mm, an inner diameter of 9 mm, and a braid angle of π/6 rad in the equilibrium state.^[^ ^37^ ^]^

### 2.2 Dynamics

Figure 1C shows the typical behaviors of each sensor value used in PRC. The input value *u*(*t*) represents the following piecewise constant periodic signal:

(1) $$\begin{array}{ccc} u(t) & = & u_{n}\;\;\left(n=\left\lfloor t/\tau \right\rfloor \right) \end{array}$$

(2) $$\begin{array}{ccc} u_{n} & = & A{\hat{u}}_{n}+B \end{array}$$

(3) $$\begin{array}{ccc} {\hat{u}}_{n} & = & \sin\frac{2\pi \tau }{T}n \end{array}$$

where *A* and *B* are the input magnitude and bias, respectively, which tune the input to a suitable range for the device. τ is the input interval, and *T* is the period of input. The present study used the following input parameters: *A* = 0.2 MPa, *B* = 0.3 MPa, τ = 0.1 s, and *T* = 1.2 s. Sensor responses are presented in Figure 1C. Here, sensor values plotted in Figure 1C were measured at the timing of updating the next input signal. The measured pressure showed nearly the same behavior as the control pressure. Furthermore, length and resistance exhibited different curves between the compression and extension phases and possessed the nonlinearity and hysteresis for the input. The load value barely responded to the control pressure and was likely a noise signal.

### 2.3 Bifurcations of Electrical Resistance Through External Load

**Figure 2:**
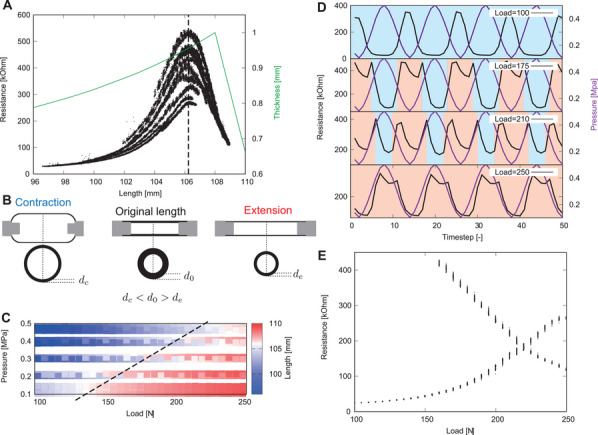
Intrinsic bifurcations of pneumatic artificial muscle. A) The graph plot of length versus resistance. B) The schematic illustration of the thickness change. C) Color map of the length for control load and pressure. The blue and red regions correspond to the contraction and extension phases in PAM, respectively. D) Resistance and pressure time series in four load conditions. The background colors (red and blue) respectively represent whether the PAM is in the contraction or extension phases in C. The load condition 100 N in the top time series is always in the contraction phase, the load conditions 175 and 210 N in the middle time series in the contraction and extension‐mixing phase, and the load condition 250 N in the bottom time series is always in the extension phase. E) The bifurcation diagram of control load versus local minimum resistance.

The behavior of the electrical resistance of the rubber changed drastically through the external load and explained the mechanics of these bifurcations. This analysis focused on alterations in the rubber's thickness induced by varying external loads applied to the PAM Specifically, the external load added to the PAM changed by 5 from 100 to 250 N under the periodic input signal, as depicted in Figure 1C and represented by Equation (1). The relationship between length and resistance is presented in **Figure** **2**A. Notably, the resistance response exhibited an oppositional trend beyond the 106 mm mark, approximately corresponding to the PAM's equilibrium length (*d*_0_ = 108 mm). Thus, these regions can be considered to correspond to the contraction and expansion phases. The length gap between 106 and 108 mm is considered to be the result of offset measurements. The electrical resistance of a rubber tube has the same tendency as the rubber thickness.^[^ ^38^ ^]^ Rubber thickness was derived from measurements of diameter and the conservation of volume. The thickness model is provided in the experimental section. The thickness of the rubber peaks at the equilibrium length, as depicted in Figure 2A. In the expansion phase, the thickness *d*_*e*_ thinned out from the equilibrium thickness *d*_0_ because the inner diameter did not change, as shown in Figure 2B. In the contraction phase, the thickness *d*_*c*_ thinned out from *d*_0_ because of expansion in the radial direction. Therefore, the resistance peaked at the equilibrium length.

Figure 2C illustrates the response of length to the applied load and pressure in the experiment. Using this method, three load regions were identified: compression phase alone, compression and extension mixing phase, and extension phase alone. The typical resistance time series of each phase is presented in Figure 2D. Resistance in the compression phase responded to the anti‐phase pressure value. Resistance in the mixing phase changed to a two‐peak behavior. In contrast to the compression phase, resistance in the extension phase responded to the pressure value in‐phase. Figure 2E depicts the bifurcation diagram, where local minimum values of resistance are plotted for the applied load values. Bifurcations were confirmed, revealing that the local minimum value changed from 1 to 2 at 160 N and became 1 again at 220 N. It is generally acknowledged in the literature that other continuous‐time dynamical systems, such as the Lorenz system, exhibit the discontinuous changes of a period of stable periodic solution, and they are seen as a bifurcation.^[^ ^39^ ^]^ These bifurcation points correspond to the change points of the compressing, mixing, and extension phases.

### 2.4 Computing Scheme

**Figure 3:**
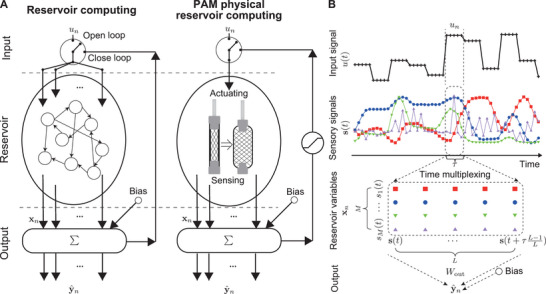
A) Schematics of physical reservoir computing. The left‐hand side depicts the schematics of reservoir computing using a neural network. The right‐hand side depicts the schematics of PRC for a pneumatic artificial muscle. B) Schematics showing the time‐multiplexing scheme to prepare reservoir states.

**Figure** **3** illustrates the computing scheme of PAM PRC. The input signal was injected as a control pressure, which is a 1D value. The PAM acted as a physical reservoir by providing a nonlinear historical response to the input. We obtained reservoir variables by sensing these responses and constructing the output values from a weighted sum of reservoir variables and a bias term.

The input signal is a piecewise constant 1D signal, which is represented in Equation (1). The nonlinearity and memory of the physical reservoir can be adjusted by tuning the input magnitude *A* and input interval τ in Equation (1) (as discussed in the Supporting Information). Sensor values at time *t* are represented in the form **s**(*t*) = (*s*_1_(*t*), ⋅⋅⋅, *s*_*M*_(*t*)), where *M* is the number of sensor values used as the reservoir variables. We obtained the reservoir variable *x*_*n*_, which corresponds to input *u*_*n*_ and is based on sensing *L* times from input injected time *t* to input updated time *t* + τ. The following equation presents $x_{n}\in R^{ML+1}$:

(4) $$\begin{array}{c} x_{n}=\left[s\left(t\right);s\left(t+\tau \frac{1}{L}\right);\cdots ;s\left(t+\tau \frac{L-1}{L}\right);1\right] \end{array}$$

where the number of samples is represented by *L*. This multiplexing method,^[^ ^40^ ^]^ which is known as time‐multiplexing, boosts the computational power of the reservoir from a small number of variables This method has been widely used in PRC.^[^ ^8^, ^41^, ^42^ ^]^ In the present study, unless specified otherwise, we will use *L* = 5. The output values ${\hat{y}}_{n}=\left({\hat{y}}_{n,1},\text{…},{\hat{y}}_{n,K}\right)\in R^{K}$ were generated as follows:

(5) $$\begin{array}{c} {\hat{y}}_{n}=W_{\mathrm{out}}^{T}x_{n} \end{array}$$

where $W_{\mathrm{out}}\in R^{(ML+1)\times K}$ is the output weight (+1 in the index means a bias term). The output weight is obtained by ridge regression:

(6) $$\begin{array}{c} W_{\mathrm{out}}={\left(X^{T}X+\lambda I\right)}^{-1}X^{T}Y \end{array}$$

where $X=\left(x_{N_{\mathrm{wash}}+1}\cdots x_{N_{\mathrm{wash}}+N_{\mathrm{train}}}\right)\in R^{\left(ML+1\right)\times N_{\mathrm{train}}}$ is the training data matrix, $Y=\left(y_{N_{\mathrm{wash}}+1}\cdots y_{N_{\mathrm{wash}}+N_{\mathrm{train}}}\right)\in R^{K\times N_{\mathrm{train}}}$ is the target data matrix, and λ is the ridge parameter. The numbers of the washout and training data are represented as *N*_wash_ and *N*_train_, respectively.

The open‐ and closed‐loop settings are depicted in Figure 3A. Open‐loop represents a case in which the input signal *u*_*n*_ is external to the reservoir. Closed‐loop represents the case in which the input signal *u*_*n*_ is the output value of the reservoir one time step prior. In the closed‐loop setting, the input values, which are control pressures, are restricted to a certain range [*u*_min_, *u*_max_] to prevent the breakdown of the system.

(7) $$\begin{array}{ccc} {\hat{u}}_{n} & = & Ay_{n-1}+B \end{array}$$

(8) $$\begin{array}{ccc} u_{n} & = & \left\{\begin{array}{cc} u_{\min} & \left({\hat{u}}_{n}<u_{\min}\right)\\ {\hat{u}}_{n} & \left(u_{\min}\leq {\hat{u}}_{n}\leq u_{\max}\right)\\ u_{\max} & \left({\hat{u}}_{n}>u_{\max}\right) \end{array}\right. \end{array}$$

The present study used the value (*u*_min_, *u*_max_) = (0 MPa, 0.5 MPa). We also show the computing schematics as a pseudo‐code in the experimental section.

### 2.5 Information Processing Capacity

We investigated the information processing capabilities of each sensor value in the PAM, specifically focusing on their nonlinearity and memory To assess these capabilities, we employed the information processing capacity (IPC)^[^ ^43^ ^]^ criteria, which describes the function that the dynamical system, serves for an input signal from independent and identically distributed (i.i.d.) random variables.^[^ ^44^ ^]^ Memory and the linear/nonlinear transformation capability of the reservoir can be obtained by checking the bases of this function. IPC‐limited linear components are called memory capacity.^[^ ^45^ ^]^ Detailed definitions and formulations of the IPC are provided in the experimental section. IPC restricted the delay to ⩽*D* and the degree of polynomial functions to ⩽*K*, as IPC[*D*, *K*]. IPC[*D*, *K*] can be decomposed by function reconstruction capacities C[Y], where Y is an orthogonal basis function in the focusing functional space. The IPC[1, *K*] is referred to as memory capacity. Here, a linear component implies that total capacities integrate all the linear capacities. A higher‐order component implies that total capacities integrate all the capacities with the same degree. For a number *N* of the linear independent states of the reservoir variable, the following theoretical equation holds:^[^ ^43^ ^]^

(9) $$\begin{array}{c} \lim_{D,K\to \infty }\mathrm{IPC}[D,K]\leq N \end{array}$$

where equality is established if the reservoir has the echo state property (ESP). Here, ESP, which is an important property for reservoir computing, guarantees the reproducibility of the computing results.^[^ ^46^ ^]^

First, we showed the IPCs of each single sensor value and multiplexing sensor values in the PAM, which included pressure, length, resistance, load, and all sensors combined and time‐multiplexed. The IPCs are presented in **Table** **1**. The IPC of the pressure was nearly 1, and the pressure could be completely described by the input sequence. Therefore, the pressure could be a computational node that has the ESP for the input. The IPCs of length and resistance were slightly lower than 1, and these sensor values could nearly be described by the input sequence; however, they had few irreproducible components. The IPC of the load was 0.0037 and nearly 0, and the load moved nearly independently of the input. The IPC could be improved to ≈10 by combining all types of sensors and using time‐multiplexing. The total number of reservoir variables was 20 = 4 × 5. An IPC lower than the number of variables implied the existence of input‐independent or linearly dependent components.

**Table 1:** Major capacities of sensors.

Target function	Capacity [‐]				All sensors
	Pressure	Length	Resistance	Load	
IPC	0.993	0.975	0.943	0.004	9.861
$P_{1}\left(u_{n}\right)$	0.873	0.369	0.509	0.004	0.957
$P_{1}\left(u_{n-1}\right)$	0.089	0.527	0.223	0.000	0.996
$P_{1}\left(u_{n-2}\right)$	0.000	0.026	0.013	0.000	0.769
$P_{2}\left(u_{n}\right)$	0.007	0.002	0.054	0.000	0.555
$P_{1}\left(u_{n}\right)P_{1}\left(u_{n-1}\right)$	0.006	0.012	0.095	0.000	0.340
$P_{2}\left(u_{n}\right)P_{1}\left(u_{n-1}\right)$	0.036	0.009	0.018	0.000	0.373

**Figure 4:**
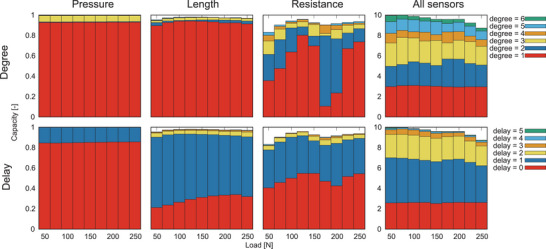
Information processing capacities of pneumatic artificial muscle sensory values. The bars indicate the decompositions of the IPCs through the degree components and memory components (The method for the decomposition is provided in the Experimental Section). The length and resistance graphs share a common vertical axis coordinate with the pressure graph.

Further details, such as the nonlinear and memory capacities of the IPCs in the PAM, were examined. **Figure** **4** depicts the dependency of the IPCs on the external load. The capacities of the pressure did not change through external load. The capacities with a delay 0 in the length monotonically increased as the external load increased from 50 to 250 N. Therefore, a PAM with a smaller external load could produce information processing that requires more memory. However, the degree components in the resistance non‐monotonically changed through the external load. In addition, the linear components exhibited a monotonically increasing trend as the external load rose up to 150 N. At 175 and 200 N of external load, the degree two components became predominant, while the linear components regained dominance when the external load exceeded 225 N. The transitions between increasing and decreasing trends aligned with the bifurcation points of resistance, indicating that these critical behaviors stemmed from intrinsic resistance bifurcations.

### 2.6 Length Sensor Emulation

We evaluated the performance of PAM PRC in an open‐loop setting. First, we investigated PAM length sensor emulation,^[^ ^37^ ^]^ which is a practical task that emulates the PAM length sensor value from the input pressure value. A laser displacement sensor, which is a standard length sensor for the PAM, is made of a rigid component that reduces the softness of the PAM. Therefore, emulating the length sensor using other sensory values constitutes an important method to ensure softness. Although the length dynamics of the PAM respond nonlinearly to hysteresis for the input pressure, PAM length time series can be predicted by a recurrent neural network.^[^ ^37^, ^47^, ^48^, ^49^, ^50^, ^51^ ^]^ Here, the input sequence arose from uniformly random values and was transformed to fit within the interval [0, 0.5] MPa for control pressure values, as (*A*, *B*) = (0.5, 0) in Equation (2).

Furthermore, we evaluated the performance of the task using the normalized mean squared error (NMSE), as follows:

(10) $$\begin{array}{c} \mathrm{NMSE}=\frac{\frac{1}{N_{\mathrm{eval}}}\sum_{i=1}^{N_{\mathrm{eval}}}{\left({\hat{y}}_{i}-y_{i}\right)}^{2}}{\sigma^{2}\left(y_{i}\right)} \end{array}$$

where *N*_eval_ is the number of evaluation data. In addition, we assessed PRC from the perspective of computational cost, which includes both training time and prediction time. Training time refers to the computation time required for network optimization. Prediction time pertains to the computational time necessary for generating a single data point prediction. In PRC, we only evaluated the time spent on linear summation calculations executed on an external computer, without accounting for the time required for physical actuation or sensing. The computation time evaluation employed a computer equipped with a 12th Gen Intel(R) Core(TM) i9‐12900KF CPU running at 3.20 GHz.

In the following experiments, the number for the washout data was *N*_wash_ = 1, 000, the number for the training data was *N*_train_ = 40, 000, and the number for the evaluation data was *N*_eval_ = 9, 000. We compared the performances between PAM PRC, the physical model, long short‐term memory (LSTM),^[^ ^52^ ^]^ and the echo state network (ESN),^[^ ^11^ ^]^ which is a typical recurrent neural network in reservoir computing. The formulations and parameter selections of comparison models are presented in the experimental section. We multiplexed sensor values in the PAM using time‐multiplexing *L* = 5. In one instance, we used 15 reservoir variables (which included time‐multiplexed pressure, resistance, and load). The LSTM and ESN had the same number of computational nodes as the number of reservoir variables in PAM PRC (15).

**Figure 5:**
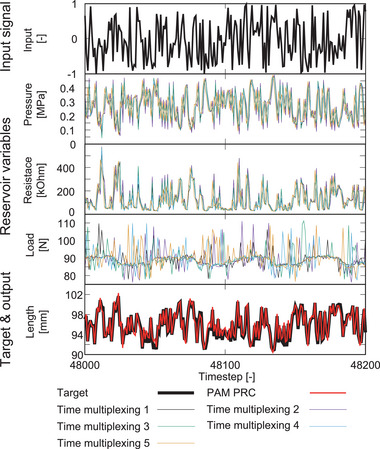
Time series of the input, reservoir variables, output, and prediction in the length sensor emulation task. The red line is the prediction signal of the PAM PRC with loads.

**Figure** **5** illustrates the time series of the input, reservoir variable, target, and output signals. The results of length sensor emulation tasks are shown in **Table** **2**. When the training data consist of a sufficient 40,000 samples, LSTM achieves the lowest NMSE, but when the training data are very small, only 100 samples, PRC exhibits the lowest NMSE, demonstrating its high generalization performance even with limited data. This aspect is an important advantage in the information processing of soft materials, as they generally have lower durability than rigid materials, and their material properties can easily change over a long time period. Regarding computational cost, PRC excels in outsourcing computations to the PAM itself. In training, LSTM incurs a substantial computational cost due to backpropagation through time. In contrast, the reservoir calculations of ESN and PRC enable batch learning in linear regression, resulting in ≈1/30th of the computational time required for execution. In the prediction phase, PRC particularly succeeds in reducing computational overhead. While LSTM and ESN require network time evolution, PRC only needs to perform linear summation. As a result, PRC achieves a computational speed roughly 10^4^ times faster than LSTM and 100 times faster than ESN.

**Table 2:** Results of length sensor emulation.

Performances	System			
	PAM physical model	LSTM	ESN	PAM PRC
		(*N* = 15)	(*N* = 15)	(*N* = 15)
NMSE [‐] (*N*_train_ = 40000)	0.0893	**0.0147**	0.0311	0.0294
NMSE [‐] (*N*_train_ = 100)	–	0.0307	0.0353	**0.0301**
Training time [ms]	–	144,892	5.31	**4.70**
Prediction time [ms]	0.443	0.220	3.76 × 10^−3^	**2.00 × 10^−5^**

### 2.7 Attractor Embedding

The present study analyzed closed‐loop control by PRC in PAMs. First, we analyzed the potential to embed attractors in a PAM. We focused on the limit cycle and the strange attractors of the logistic and Hénon map. The limit cycle defined by Equation (3) was a 1D periodic dynamic. These rhythm dynamics are important as a central pattern generator in robot control.^[^ ^53^ ^]^ In addition, chaotic oscillators, in addition to periodic ones, hold significance in robot control. As a central pattern generator, a chaotic oscillator can derive adaptive and exploratory behaviors by its complex dynamics.^[^ ^30^, ^31^ ^]^ The logistic map, which is a 1D dynamical system with discrete time, was defined by the following equation:

(11) $$\begin{array}{c} y_{n+1}=ay_{n}\left(1-y_{n}\right) \end{array}$$

where *a* is a model parameter and is set as the chaotic parameter *a* = 3.7. The embedding of logistic dynamics does not require memory because the next step of the logistic map can be determined only by the current step. In addition, we conducted a closed‐loop task for a more complicated chaotic attractor in the Hénon map using two parallel PAMs as the physical reservoir. The Hénon map is a two‐dimensional discrete‐time dynamical system, which is defined as follows:

(12) $$\begin{array}{ccc} y_{n+1}^{1} & = & 1-a{\left(y_{n}^{1}\right)}^{2}+y_{n}^{2} \end{array}$$

(13) $$\begin{array}{ccc} y_{n+1}^{2} & = & by_{n}^{1} \end{array}$$

where *a* and *b* are the model parameters and are set as (*a*, *b*) = (1.4, 0.3), which show chaos. Here, we used $y_{n}^{1}$ as the closed‐loop feedback signal (so ${\hat{u}}_{n+1}=y_{n}^{1}$) for both of the PAMs. To prepare reservoir variable **x**_*n*_, we used sensory variables **s**_1_(*t*) and **s**_2_(*t*), which correspond to each PAM as follows:

(14) $$\begin{array}{c} x_{n}=\left[s_{1}\left(t\right);\cdots ;s1\left(t+\tau \frac{L-1}{L}\right);s_{2}\left(t\right);\cdots ;s_{2}\left(t+\tau \frac{L-1}{L}\right);1\right] \end{array}$$

The two PAMs had the same physical configuration but with different external loads, 100 and 200 N, so the sensory responses **s**_1_(*t*) and **s**_2_(*t*) to the same input pressure were different. This type of reservoir computing setup is referred to as spatial multiplexing.^[^ ^54^ ^]^ The spatial multiplexing setup provides a further number of nodes and repertory of computational capabilities for the physical reservoir. We used this method to demonstrate the potential of our framework to embed more complicated attractors, namely the Hènon attractor in this section and the Van der Pol oscillator and Rössler attractor^[^ ^55^ ^]^ in the Supporting Information.

In the training phase, we injected *y*_*n*_ into the reservoir using an open‐loop and trained the output weight using *y*_*n* + 1_ as a teacher signal (this training scheme is known as teacher forcing^[^ ^56^ ^]^). The input range of all experiments was set to [0.1,0.5] MPa by tuning *A* and *B* in Equation (1). Furthermore, the input interval of the PAM control pressure was set as τ = 0.1, 0.2, and 0.3 s, and the number for time‐multiplexing were *L* = 5, 20, and 30 in the experiments for the limit cycle, the logistic map, and the Hénon map, respectively. In all of the experiments, we fixed the number of washout data at *N*_wash_ = 1, 000 and the number of training data at *N*_train_ = 4, 000. In the prediction phase, we switched the open‐loop and closed‐loop after 1,000 time steps. In addition, we have evaluated the embedding results using the output time series, the attractor in the delayed coordinate system, and power spectra.

**Figure 6:**
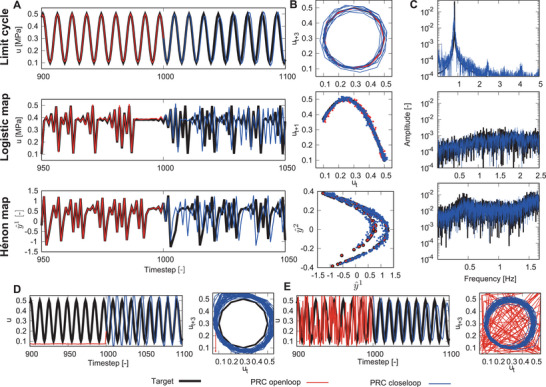
Results of the closed‐loop at the attractor embedding. A) Time series of the target and PRC output signals. B) Attractors of the target and PRC output. C) Fourier spectra of the target and PRC output. D) Time series and attractor in the limit cycle, embedding from 0 inputs. E) Time series and attractor in the limit cycle, embedding from a random input.

**Figure** **6** illustrates the outcomes of the attractor embedding. We analyzed the embedding performance for aspects of short‐term traceability, attractor similarity, and Fourier spectrum similarity, visually presented in Figure 6A–C, respectively. In limit cycle embedding, the prediction signal traces the target signal and attractor, and the Fourier spectrum of the prediction has the same peak as the target Fourier spectrum. In logistic map embedding, the output time series deviated from the target signal as 6‐time steps passed after switching from an open‐ to a closed‐loop because of the initial state sensitivity of chaos. Nevertheless, the output signal traces the target attractor and Fourier spectra with a broad band induced by chaotic dynamics. We quantitatively evaluated these three embedding performances using criteria in the Supporting Information and compared them with artificial neural networks. As a result, the PAM PRC has comparable performance with an ESN with 20 in the task of attractor embedding for the logistic map. In Hénon attractor embedding, even though there was only one variable feedback input for the reservoir, it was possible to embed a two‐dimensional dynamical system by using the reservoir's memory.

Next, we evaluated the robustness of attractor embedding. For this, we injected a random signal from the target signal and confirmed that the output signal could quickly return to the target attractor after switching to a closed loop. We focused on the limit cycle as a target attractor, and the input signals in the open‐loop were 0 and random signals. The results are depicted in Figure 6D,E. The output signals quickly returned to the target attractor after switching.

### 2.8 Bifurcation Embedding

We previously confirmed that the IPC of the resistance in the PAM could undergo significant changes by adjusting the external load in an open‐loop setting. Moreover, we found the change in the output signal of PAM PRC through the external load in a closed‐loop setting. The following training data were used:

a) A limit cycle with a period of 1.2 s with an external load of 100 N (same as the limit cycle in Figure 6);
b) A limit cycle with a period of 1.2 s with external loads of 100 N and 250 N;
c) Limit cycles with periods of 1.2 and 2.4 s with external loads of 100 and 250 N, respectively;
d) The chaotic trajectory of the logistic map, where *a* = 3.7, with an external load of 100 N (same as in the case of the logistic map in Figure 6)
e) The period 4 trajectory of the logistic map, where *a* = 3.55 with an external load of 100 N.

We confirmed the change in the output signal in the closed‐loop control when the external load changed by 5 from 100 to 250 N at every 2,000 time steps.

**Figure 7:**
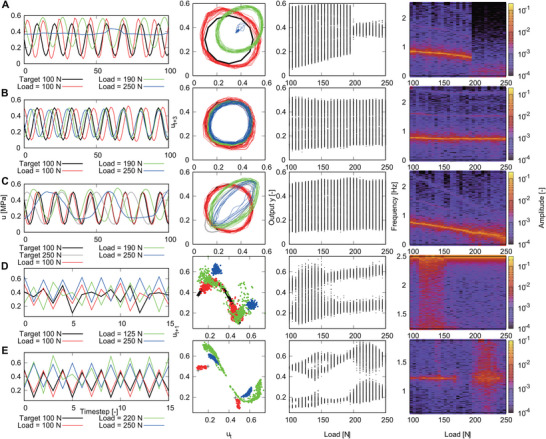
Bifurcation embedding using closed‐loop control. A) Training the sinusoidal wave when the external load is 100 N. B) Training the sinusoidal wave when the external loads are 100 and 250 N. C) Training sinusoidal waves with different periods when external loads are 100 and 250 N. D) Training the strange attractor of the logistic map when the external load is 100 N. E) Training period 4 trajectory when the external load is 100 N.

**Figure** **7** depicts the results. In experiment A, the amplitude and frequency of the limit cycle continuously changed in the range from a load of 100 to 200 N. However, the limit cycle structure of the output signal suddenly collapsed at an external load of 200 N, and the output signal changed to nearly static dynamics. This switching point was around the second bifurcation point of the resistance, as shown in Figure 2D. Thus, the dynamics may switch because the bifurcation of the resistance propagated to the entire dynamics of the PAM via closed‐loop control. Conversely, the results of experiment B indicated that it is possible to suppress the closed‐loop bifurcations. In experiment B, we trained the limit cycle when external loads were 100 and 250 N. The bifurcation structure that appeared in experiment A did not occur, and PAM with all external load conditions from 100 to 250 N generated the same limit cycles as the output. When comparing elements of output weights *W*_out_ of experiment B and experiment A, it was observed that the elements of weights corresponding to resistance values in experiment B were more than 10^−7^ times smaller than in experiment A (see Supporting Information). Therefore, in experiment B, the learning automatically avoided utilizing the resistance values as reservoir variables that dramatically change information processing capabilities through the external load shown in Figure 4, and the closed‐loop bifurcation could be suppressed. In experiment C, we trained limit cycles with different frequencies when external loads were 100 and 250 N. The results revealed that the frequency of the closed‐loop dynamics with intermediate external loads was linearly interpolated.

The results of experiments D and E revealed that periodic and chaotic dynamics could be embedded simultaneously and that one of the dynamics could be generated from learning another dynamics. In experiment D, we trained chaotic dynamics in the logistic map when the external load was 100 N. The dynamics switched from chaotic dynamics to period 2 dynamics when the external load was 170 N, which corresponded to the first step of the bifurcation of resistance. As the bifurcation diagram indicates, period 2 dynamics appeared intermittently, acting as a window for period‐doubling bifurcation. In experiment E, we trained period 4 dynamics in the logistic map when the external load was 100 N. The dynamics switched to chaotic dynamics with a one‐dimensional attractor in the delay coordinate and broad spectra when there was an external load of 200 N, which corresponded to the second bifurcation of the resistance. The chaotic attractor in the delay coordinate had an alternative shape, similar to a cubic function. In addition, the dynamics had an unstable fixed point near *y*_*t* + 1_ = *y*_*t*_, as there was a hole at the intersection of *y*_*t* + 1_ = *y*_*t*_ and the attractor.

These bifurcation embedding results could be useful for robotics applications. For instance, the automatic switching conducted in experiment A could be used for an emergency stop when the external load exceeds the threshold and an idling stop that transitions to a stationary state while the main power is on. This presents the possibility of internalizing adaptive behavioral control that depends on changes in the environment. In addition, the results of experiments B and C revealed that this switching can be turned off by explicit training on both sides of the bifurcation of the inherent dynamics. Furthermore, the results of experiments D and E have demonstrated that multiple qualitatively different dynamics, including chaos, could be switched according to changes in the environment. The results of experiments D and E did not indicate the desired bifurcation structure but instead showed a bifurcation structure based on training data and reservoir dynamics. We present the embedding of the bifurcation structure, which includes the desired qualitatively different signals, by training dynamics on both sides of bifurcation explicitly in the Supporting Information.

## 3 Conclusion

We have demonstrated that PRC's architecture, with nonlinearity and memory in PAM as a physical reservoir, allows it to perform various tasks, including self‐motion estimation, closed‐loop control, and bifurcation embedding. These findings shed light on the potential, constraints, and avenues for future exploration in computational capabilities integrated within a robotic body.

In the PAM length sensor emulation, we have confirmed that PAM PRC exhibits excellent predictive performance even with a limited amount of training data. Additionally, we have verified that PRC can execute with very low computational cost by outsourcing computations to the physical dynamics. In the context of PAM, where flexibility is crucial, the ability to learn before material properties change and the minimization of external computing devices that may compromise softness are both highly important. Therefore, PRC, with these advantages, can be considered a valuable approach for information processing in PAM.

We demonstrated that a PAM can be embedded in qualitatively different attractors from the training attractor in the closed‐loop experiments. These results suggest that bifurcations in the morphology may have the potential to be exploited to embed the bifurcation structure of the targeted dynamical system. However, the mechanism to embed the bifurcation structure into the reservoir has not been fully understood to date.^[^ ^35^ ^]^ Moreover, the necessity of the intrinsic bifurcations of the reservoir for bifurcation embedding remains unknown.

This bifurcation embedding into the body suggests the strong potential of robot control. For instance, if we can embed the period‐doubling bifurcation in the morphology of the robot, it may be possible for the robot to generate all the arbitrary periodic dynamics and chaos underlying Li–Yorke chaos^[^ ^57^ ^]^ from learning only finite period patterns.

There are some important notes and limitations to consider in the present study. Nonlinearity and memory take various forms beyond the physical reservoir. For instance, during actuation, variations in input delay and input intervals may function as memory. In sensing, physical quantities are treated as values with finite precision and incorporate nonlinear cutoffs. In feedback control, similar cutoffs, as in Equation (8), are applied to match the input's specifications. PRC implicitly leverages these information processing capabilities in its computations. However, from an engineering viewpoint, actively utilizing the information processing capabilities of devices accompanying the physical reservoir is considered beneficial.

Artificial recurrent neural networks with sufficient size and training data outperform PAM PRC in the length sensor emulation task and closed‐loop chaotic attractor embedding of the logistic map (the quantitative analyses for attractor embedding of the logistic map are provided in the Supporting Information). Although morphological computation does not aim to achieve a universal computer with extensive computational capabilities, it seeks to leverage existing computational capabilities in the body. In this context, while a PRC may have fewer computational capabilities than an external computer, it can substantially reduce computational and communication costs associated with externally attached devices.

The present study has indicated that the body dynamics of PAM have high computational capability. We believe that these results can be expanded to practical situations. Based on the results of Hénon attractor embedding using spatial multiplexing, the structures consisting of multiple PAMs, such as a robot arm and wearable assistance suit, may have the potential to embed higher‐dimensional and more complex dynamics than single PAMs. For example, if we can embed chaotic itineracy into the robot's body, the robot can switch between numerous primitive patterns autonomously and randomly.^[^ ^58^ ^]^ Moreover, the embedded bifurcation structure could serve as an adaptive pattern switch for the environment, such as for anomaly detection and robot failure prevention, because the bifurcation points correspond to the change points of the dynamic phase of the body dynamics, such as the contraction and extension phases in the PAM. These potentials of dynamical systems can provide valuable insights into various forms of PRC, including neuromorphic,^[^ ^41^, ^42^, ^59^, ^60^ ^]^ natural,^[^ ^61^ ^]^ and biological computing.^[^ ^62^, ^63^ ^]^ By harnessing these dynamics, it may be possible to extract even more advanced functionalities from such systems.

## 4 Experimental Section

### Experimental Hardware and Software

The PAMs used in this study are the same as in ref. [25]. The rubber tube consists mainly of isoprene polymer, carbon black as a reinforcing filler, sulfur as a curing agent, and oil to improve processability. The detailed material properties, including the stress‐strain curve, are provided in ref. [25]. The information on the experimental hardware and software was also provided, which are shown in Figure 2A,B, in **Table** **3**.

**Table 3:** Experimental hardware and software.

System	Product
Laser displacement meter	OPTEX FA Corp. CD22‐100V2
Resistance meter	Agilent Technologies Corp. A34411A / Applying a constant voltage source system
Air pressure control (PAM and load)	CKD Corp. Electropneumatic regulator EVR2500
Pneumatic cylinder	SNC MQQTB40
Control system	LabVIEW

In the experiments on attractor embedding for the logistic and Hénon maps shown in Figure 6, the constant voltage source‐based measurement system was applied for the resistance measurement to improve the reproducibility and frequency of measurement. In this system, 5 V was applied to the rubber tube using a constant voltage source, the reference resistor and PAM were placed in a series, and the resistance of the PAM was calculated from the respective voltages. The multimeter of Agilent was used in all other experiments in the main text.

### Pneumatic Artificial Muscle Thickness Model

The thickness model of the PAM is presented in Figure 2A. Thickness was calculated using the following equation:

(15) $$\begin{array}{c} d=R-r \end{array}$$

where it was assumed that the rubber tube in the PAM is a uniform cylinder and that *R* and *r* are the outer and inner radius of the cylinder, respectively. Furthermore, the below linear relationship between the length and outer radius was assumed because the coefficient of determination between them was 0.9934, which was obtained from the nine values of length and thickness of the rubber tube with an external load of 50 N.

(16) $$\begin{array}{c} R=-0.3382l+47.525 \end{array}$$

where the length of the rubber tube is represented by *l*. The inner radius *r* was obtained using the following equation because of the constraint of the constant volume of rubber and restriction of both ends of the tube:

(17) $$\begin{array}{c} r=\left\{\begin{array}{cc} r_{0} & \left(l\geq l_{0}\right)\\ \sqrt{R^{2}-\frac{V}{l\pi }} & \left(l<l_{0}\right) \end{array}\right. \end{array}$$

where *V* is the volume of rubber and *r*_0_ and *l*_0_ are the inner radius and length in the equilibrium length, respectively. Figure 2A presents the length and thickness that were calculated using the above equations.

### Pseudo‐Code of PRC

Here, the pseudo‐code was provided, which corresponds to Equations (4) and (5) Algorithm 1.

Algorithm 1 **Calculation of the output $\hat{y}$ from sensory time series s(*t*) in PRC**

**Table:** 

**for** *i* in [1, *L*] **do**
**for** *j* in [1, *M*] **do**
*x* append *s*_*j*_(*t* + τ(*j* − 1)/*L*)
**end for**
**end for**
*x* append 1
$\hat{y}=W_{out}\cdot x$

### Echo State Network

The architectures of the ESN were compared with PAM PRC. The *i*th computational node at time *t* is represented as $x_{t}^{i}$, the *j*th input node is represented as *u* ^*j*^, and the *l*th output node at time *t* is represented as ${\hat{y}}_{t}^{l}$. The computational nodes and outputs of the ESN are given by the following:

(18) $$\begin{array}{ccc} x_{t}^{i} & = & f\left(A_{\mathrm{cp}}\sum_{j=1}^{N}w_{ij}x_{t-1}^{j}+A_{\mathrm{in}}\sum_{j=1}^{N}w_{ij}^{\mathrm{in}}u_{t}^{j}+b\right) \end{array}$$

(19) $$\begin{array}{ccc} {\hat{y}}_{t}^{l} & = & \sum_{i=0}^{N}w_{i,j}^{\mathrm{out}}x_{t}^{i} \end{array}$$

where the activation function is given by *f*, which is the hyperbolic tangent. Each node of the input weight $W_{\mathrm{in}}=\left(w_{ij}^{\mathrm{in}}\right)$ comprises a uniform distribution with [− 1, 1]. Each node of the internal weight *W* = (*w*_*ij*_) comprises a uniform distribution with [− 1, 1] and is normalized to make the spectral radius 1. Each node of the bias **b** = (*b*_1_⋅⋅⋅*b*_*N*_)^⊤^ comprises a uniform distribution with [− 1, 1]. The coupling magnitude *A*_cp_ coincides with the spectral radius of *A*_cp_ *W*. The bias term $x_{t}^{0}$ is set as $x_{t}^{0}=1$. The output weight $W_{\mathrm{out}}=\left(w_{ij}^{\mathrm{out}}\right)$ is tuned by training. We fix *A*_in_ = 1 and optimize *A*_cp_ by grid search for the interval [0, 1.2] in each task.

### Long Short‐Term Memory

LSTM was implemented using the PyTorch library. While basing the parameters on a study^[^ ^51^ ^]^ that also used LSTM for PAM length sensor emulation, experiments were conducted using the parameter set outlined in **Table** **4**.

**Table 4:** Hyperparameters in LSTM.

Hyperparameter	Value
Number of computational nodes	15
Optimizer	ADAM^[^ ^64^ ^]^
Learning rate	0.001
Batch size	500
Number of epochs	500
Input time window	4

### Dynamical Model of the PAM

The length dynamics of the PAM was estimated from the injected pressure and load for the control. The models of PAM dynamics were widely investigated in previous studies.^[^ ^65^, ^66^ ^]^ Based on these studies, the following length model of the PAM was used:

(20) $$\begin{array}{c} M\ddot{x}=-F_{\mathrm{elas}}\left(x\right)-F_{\mathrm{fric}}\left(\dot{x}\right)-F_{\mathrm{pre}}\left(x,p\left(t\right)\right)+F_{\mathrm{ex}}\left(t\right) \end{array}$$

where the displacement of the PAM length is represented as *x* and the mass of the PAM is represented as *M*; the elastic force of the rubber, friction of the rubber, and tension of volume change by pressure are represented as *F*_elas_(*x*), $F_{\mathrm{fric}}\left(\dot{x}\right)$, and *F*_pre_(*x*, *p*(*t*)), respectively; and the input pressure and input load are represented as *p*(*t*) and *F*_*ex*_. Here, tension by pressure is derived from the following Schulze equation:

(21) $$\begin{array}{c} F_{\mathrm{pre}}\left(x,p\left(t\right)\right)=\frac{\pi D_{0}^{2}p\left(t\right)}{4}\frac{1}{\sin^{2}\theta_{0}}\left[3{\left(1-\varepsilon \right)}^{2}\cos^{2}\theta_{0}-1\right] \end{array}$$

where the strain of the PAM is represented as ε = (*l*_0_ − *x*)/*l*_0_ and the equilibrium length, inner radius, and angle of the braided cord are represented as *l*_0_, *D*_0_, and θ_0_, respectively. Note that the Schulze equation assumes that the PAM is a uniform cylinder with 0 thickness. However, when the real PAM is compressed, it is not a cylinder but instead becomes a bent shape because both ends of the PAM are fixed. A model that considers the non uniform and bent shape of the PAM was previously proposed in the literature.^[^ ^66^ ^]^ It was ensured that the linear elasticity *F*_elas_(*x*)∝*x* is the elasticity of the PAM. The length of the PAM was could be accurately estimated by solving the equation of the equilibrium of *F*_pre_(*x*), *F*_elas_(*x*), and *F*_*ex*_ in the static state. However, in the dynamic state, in which the PAM continues to move, it was difficult to estimate the PAM dynamics because the Schulze equation cannot consider the hysteresis depicted in Figure 2 in the main text. The causes of the hysteresis may be the effects of the Coulomb and viscous frictions.^[^ ^67^, ^68^, ^69^ ^]^ Therefore, Equation (20) could be rewritten using the following equation:

(22) $$\begin{array}{c} \ddot{x}=-Ax-B\dot{x}-C\mathrm{sgn}\left(\dot{x}\right)+D\left(-F_{\mathrm{pre}}\left(x,p\left(t\right)\right)+F_{\mathrm{ex}}\left(t\right)\right) \end{array}$$

Here, *A*, *B*, *C*, and *D* are the parameters of the model. These parameters were optimized using grid search in the range of *A* ∈ [1, 000, 10, 000], *B* ∈ [10, 100], *C* ∈ [10, 100], and *D* ∈ [0.1, 1.0], and the parameters used in this experiments were (*A*, *B*, *C*, *D*) = (6353, 80.05, 10, 0.635). The measured length values were offset because of a measurement error; thus, a bias was added to the length‐predicting value from the physical model to coincide with the average values of the measured and predicted lengths.

### Calculation Method of Information Processing Capacity

Here, the detailed definition of IPC^[^ ^43^ ^]^ and the setup of IPC experiments were described. It was assumed that the space of the input, reservoir, and output is $R^{1}$, $R^{N}$, and $R^{1}$, respectively. The input, reservoir, output, and target time series were represented as $U=(\text{…},u_{t-1},u_{t},\text{…})$, $X=(\text{…},x_{t-1},x_{t},\text{…})$, $\hat{Y}=\left(\text{…},{\hat{y}}_{t-1},{\hat{y}}_{t},\text{…}\right)$, and $Y=(\text{…},y_{t-1},y_{t},\text{…})$, respectively.

The capacity of the reservoir time series X for a target time series Y is defined by the following equation:

(23) $$\begin{array}{ccc} C[X,Y] & = & 1-\frac{\min_{W_{\mathrm{out}}}\left\langle {\left(y_{t}-{\hat{y}}_{t}\right)}^{2}\right\rangle }{\left\langle u_{t}^{2}\right\rangle }\\ & = & \frac{{\left\langle y_{t}x_{t}\right\rangle }^{⊤}{\left\langle x_{t}x_{t}^{⊤}\right\rangle }^{-1}\left\langle y_{t}x_{t}\right\rangle }{\left\langle y_{t}^{2}\right\rangle } \end{array}$$

where $\left\langle y_{t}^{2}\right\rangle$ denotes the average of $y_{t}^{2}$ through time *t*. 0≤C[X,Y]≤1 always holds. When C[X,Y]=0, the reservoir never reconstructs target Y, but when C[X,Y]=1, the reservoir can completely reconstruct target Y.

If the input series comprises i.i.d. uniformly random values with range [− 1, 1], an arbitrary input echo function $y\left(U\right)\in R^{N}\to R$ can be decomposed into the following orthogonal polynomials: $g_{t}^{l}\left(U\right)\;\left(l=1,2,\text{…}\right)$, which is

(24) $$\begin{array}{c} g_{t}^{l}\left(U\right)=\prod_{d=0}^{\infty }P_{k_{l,d}}\left(u_{t-d}\right) \end{array}$$

where $P_{k}\left(u\right)$ is the Legendre polynomial with degree *k* as follows:

(25) $$\begin{array}{c} P_{k}\left(u\right)=\sqrt{\frac{2k+1}{2}}\frac{{\left(-1\right)}^{k}}{2^{k}!}\frac{d^{k}}{du^{k}}{\left(1-u^{2}\right)}^{k}\;\;\left(n=0,1,\text{…}\right) \end{array}$$

and $\left\{k_{l,d}\right\}\in Z_{\geq 0}\;\left(d=0,1,\text{…}\right)$ is a series of degrees for $g_{t}^{l}$. The set of orthogonal basis polynomials S(D,K) restricted by the delay of the input series was defined as ⩽*D* as *u*_*t*_, ⋅⋅⋅, *u*_*t* − *D*_, and the degree of polynomials to be less than and equal as ⩽*K*. Then, S(D,K) was defined in the form of the following equation:

(26) $$\begin{array}{c} S\left(D,K\right)=\left\{g^{l}\left(U\right)=\prod_{d=0}^{D}P_{k_{l,d}}\left(u_{t-d}\right)\in R^{N}\to R|\sum_{d=0}^{D}k_{l,d}\leq K\right\} \end{array}$$

When the target time series of the function $g_{t}^{l}\left(U\right)$ was defined as $G^{l}=\left\{\text{…},g_{t-1}^{l}\left(U\right),g_{t}^{l}\left(U\right),\text{…}\right\}$, the IPC was defined as the sum of capacities of these target time series. IPC[*X*, *D*, *K*], which restricts the delay to ⩽*D* and degree to ⩽*K*, is presented in the following equation:

(27) $$\begin{array}{ccc} \mathrm{IPC}[X,D,K] & = & \sum_{g^{l}\in S\left(D,K\right)}C[X,G^{l}] \end{array}$$

When Equation (23) was calculated from a finite amount of data $U_{T}=\left(u_{1},\text{…},u_{T}\right)$ and $X_{T}=\left(x_{1},\text{…},x_{T}\right)$, the capacity could be overestimated because of the finite‐size effects.^[^ ^43^ ^]^ To avoid this problem, the threshold and capacities that were less than the threshold were replaced by 0.^[^ ^44^ ^]^ In other words, the restricted capacity $C_{\varepsilon }\left[X_{T},Y_{T}\right]$ was used instead of $C[X_{T},Y_{T}]$:

(28) $$\begin{array}{c} C_{\varepsilon }\left[X_{T},Y_{T}\right]=\left\{\begin{array}{cc} C[X_{T},Y_{T}] & \left(C\left[X_{T},Y_{T}\right]\geq \varepsilon \right)\\ 0 & \left(C\left[X_{T},Y_{T}\right]<\varepsilon \right) \end{array}\right. \end{array}$$

ε = 4.0 × 10^−3^ was used in the IPC analyses for all sensors, as shown in Figure 4, and ε = 1.0^−3^ in the remaining IPC analyses, including those presented in the Supporting Information. These thresholds were the same level or larger values than thresholds determined by the surrogate data method.^[^ ^44^ ^]^

Here, the decomposition method of IPC used in Figure 4 in the main text was explained. It was defined ${\mathrm{IPC}}_{\mathrm{degree}=k}\left[X_{T},D,K\right]$, which was the IPC[X,D,K] restricted by degree *k*, in the following manner:

(29) $$\begin{array}{ccc} {\mathrm{IPC}}_{\mathrm{degree}=k}\left[X,D,K\right] & = & \sum_{g^{l}\in S\left(D,k\right)\setminus S\left(D,k-1\right)}C[X,G^{l}] \end{array}$$

From the definition, ${\mathrm{IPC}}_{\mathrm{degree}=k}\left[X,D,K\right]$ was a decomposition of IPC[X,D,K] that could be expressed in the following manner:

(30) $$\begin{array}{ccc} \sum_{k=1}^{K}{\mathrm{IPC}}_{\mathrm{degree}=k}\left[X,D,K\right] & = & \mathrm{IPC}[X,D,K] \end{array}$$

Next, it was defined ${\mathrm{IPC}}_{\mathrm{delay}=d}\left[X,D,K\right]$, which was the IPC[X,D,K] restricted by delay *d*, in the following manner:

(31) $$\begin{array}{c} {\mathrm{IPC}}_{\mathrm{delay}=d}\left[X,D,K\right]=\sum_{g^{l}\in S\left(D,K\right)}\frac{C[X,G^{l}]}{\#(D\left(g^{l}\right))}\min\left\{k_{l,d},1\right\} \end{array}$$

where $D(y^{l})$ is a set of delay components $D\left(g^{l}\right)=\{d\in Z_{\geq 0}|k_{l,d}\geq 1\}$ and #(·) is the number of elements in the set. By definition, ${\mathrm{IPC}}_{\mathrm{delay}=d}\left[X,D,K\right]$ is also a decomposition of IPC[X,D,K], which is expressed in the following manner:

(32) $$\begin{array}{ccc} \sum_{d=0}^{D}{\mathrm{IPC}}_{\mathrm{delay}=d}\left[X,D,K\right] & = & \mathrm{IPC}[X,D,K] \end{array}$$

In the experiments presented in Table 1 in the main text, *T* = 50, 000 sensory time series data of the PAM was used, which were injected random values from a uniform distribution with the range [0, 0.5] MPa, for the calculations of the capacities. In the experiments depicted in Figure 4 in the main text, *T* = 10, 000 sensor time series data of the PAM was used for the calculations of the IPCs. The initial 1,000 data in the time series were washout data. The max delay *D* and max degree *K* in the IPC calculation were (*D*, *K*) = (4, 5) in the single‐sensor cases and (*D*, *K*) = (5, 6) when all sensors were combined.

## Conflict of Interest

The authors declare no conflict of interest.

## Supporting information

Supporting Information

Supplemental Video 1

Supplemental Video 2

Supplemental Video 3

Supplemental Video 4

## Data Availability

The data that support the findings of this study are available in the supplementary material of this article.

## References

[1] T. McGeer , I. J. Robotic Res. 1990, 9, 62.

[2] N. Akashi , K. Nakajima , M. Shibayama , Y. Kuniyoshi , New J. Phys. 2022, 24, 013019.

[3] M. Hermans , B. Schrauwen , P. Bienstman , J. Dambre , PLoS ONE 2014, 9, 86696. 10.1371/journal.pone.0086696 PMC3908928 24497969

[4] R. H. Lee , E. A. B. Mulder , J. B. Hopkins , Sci. Rob. 2022, 7, abq7278. 10.1126/scirobotics.abq7278 36260698

[5] K. Nakajima , H. Hauser , R. Kang , E. Guglielmino , D. G. Caldwell , R. Pfeifer , in 2013 IEEE International Conference on Robotics and Automation, IEEE, Piscataway, NJ, 2013, pp. 1504–1511.

[6] K. Nakajima , H. Hauser , R. Kang , E. Guglielmino , D. G. Caldwell , R. Pfeifer , Front. Comput. Neurosci. 2013, 7, 91. 23847526 10.3389/fncom.2013.00091 PMC3705147

[7] K. Nakajima , H. Hauser , T. Li , R. Pfeifer , Sci. Rep. 2015, 5, 10487. 26014748 10.1038/srep10487 PMC4444959

[8] K. Nakajima , H. Hauser , T. Li , R. Pfeifer , Soft Rob. 2018, 5, 339. 10.1089/soro.2017.0075 PMC5995269 29708857

[9] K. Nakajima , T. Li , N. Akashi , in Robotic Systems and Autonomous Platforms, (Eds.: S. M. Walsh , M. S. Strano ), Woodhead Publishing, Cambridge 2019, pp. 181–196.

[10] W. Maass , T. Natschläger , H. Markram , Neural Comput. 2002, 14, 2531. 12433288 10.1162/089976602760407955

[11] H. Jaeger , H. Haas , Science 2004, 304, 78. 15064413 10.1126/science.1091277

[12] K. Nakajima , I. Fischer , Reservoir Computing–Theory, Physical Implementations, and Applications, Springer, Singapore 2021.

[13] K. Nakajima , Jpn. J. Appl. Phys. 2020, 59, 060501.

[14] H. Hauser , A. J. Ijspeert , R. M. Füchslin , R. Pfeifer , W. Maass , Biol. Cybern. 2011, 105, 355. 22290137 10.1007/s00422-012-0471-0

[15] K. Caluwaerts , M. D'Haene , D. Verstraeten , B. Schrauwen , Artifi. life 2013, 19, 35. 10.1162/ARTL_a_00080 23186351

[16] R. Terajima , K. Inoue , S. Yonekura , K. Nakajima , Y. Kuniyoshi , IEEE Rob. Autom. Lett. 2022, 7, 1597.

[17] Q. Zhao , K. Nakajima , H. Sumioka , H. Hauser , R. Pfeifer , presented at *2013 IEEE/RSJ International Conference on Intelligent Robots and Systems* , Tokyo, Japan, November 2013.

[18] Y. Horii , K. Inoue , S. Nishikawa , K. Nakajima , R. Niiyama , Y. Kuniyoshi , ALIFE 2021: The 2021 Conference on Artificial Life, MIT Press, Cambridge, MA 2021.

[19] H. Schulte , The Application of External Power in Proshetics and Orhotics: A Report, National Academy of Sciences, Washington DC 1961, pp. 94–115.

[20] M. Gavrilović , M. Marić , Med. Biol. Eng. 1969, 7, 77. 5795709 10.1007/BF02474672

[21] F. Daerden , D. Lefeber , Eur. J. Mech. Environ. Eng. 2002, 47, 11.

[22] D. Trivedi , C. D. Rahn , W. M. Kier , I. D. Walker , Appl. Bionics Biomech. 2008, 5, 99.

[23] T. Kanno , D. Morisaki , R. Miyazaki , G. Endo , K. Kawashima , in 2015 IEEE International Conference on Rehabilitation Robotics (ICORR), IEEE, Pistacataway, NJ 2015, pp. 565–570.

[24] D. Trivedi , D. Dienno , C. D. Rahn , J. Mech. Des. 2008, 130, 091402.

[25] R. Sakurai , M. Nishida , T. Jo , Y. Wakao , K. Nakajima , J. Rob. Mech. 2022, 34, 240.

[26] H. Hayashi , T. Kawase , T. Miyazaki , M. Sogabe , Y. Nakajima , K. Kawashima , in 2022 International Conference on Robotics and Automation (ICRA), IEEE, Piscataway, NJ 2022, pp. 3245–3251.

[27] M. Eder , F. Hisch , H. Hauser , Adv. Rob. 2018, 32, 375.

[28] H. Shinkawa , T. Kawase , T. Miyazaki , T. Kanno , M. Sogabe , K. Kawashima , in 2023 IEEE International Conference on Robotics and Automation (ICRA), IEEE, Piscataway, NJ 2023, pp. 537–543.

[29] A. Pitti , R. Niiyama , Y. Kuniyoshi , Auton. Rob. 2010, 28, 317.

[30] S. Steingrube , M. Timme , F. Wörgötter , P. Manoonpong , Nat. Phys. 2010, 6, 224.

[31] X. Zang , S. Iqbal , Y. Zhu , X. Liu , J. Zhao , Int. J. Adv. Rob. Syst. 2016, 13, 60.

[32] R. Tokunaga , S. Kajiwara , T. Matsumoto , Phys. D 1994, 79, 348.

[33] Y. Itoh , Y. Tada , M. Adachi , Nonlinear Theor. Appl., IEICE 2017, 8, 2.

[34] Y. Itoh , S. Uenohara , M. Adachi , T. Morie , K. Aihara , Chaos 2020, 30, 013128. 32013489 10.1063/1.5119187

[35] J. Z. Kim , Z. Lu , E. Nozari , G. J. Pappas , D. S. Bassett , Nat. Mach. Intell. 2021, 3, 316.

[36] M. Hara , H. Kokubu , J. Dyn. Differ. Equations 2022, 36, 515.

[37] R. Sakurai , M. Nishida , H. Sakurai , Y. Wakao , N. Akashi , Y. Kuniyoshi , Y. Minami , K. Nakajima , in 2020 3rd IEEE International Conference on Soft Rob. (RoboSoft), IEEE, Piscataway, NJ 2020, pp. 710–717.

[38] K. Yamaguchi , J. Busfield , A. Thomas , J. Polym. Sci., Part B: Polym. Phys. 2003, 41, 2079.

[39] K. T. Alligood , T. D. Sauer , J. A. Yorke , D. Chillingworth , SIAM Rev. 1998, 40, 732.

[40] L. Appeltant , M. C. Soriano , G. Van der Sande , J. Danckaert , S. Massar , J. Dambre , B. Schrauwen , C. R. Mirasso , I. Fischer , Nat. Commun. 2011, 2, 1. 10.1038/ncomms1476 PMC3195233 21915110

[41] K. Fujii , K. Nakajima , Phys. Rev. Appl. 2017, 8, 024030.

[42] J. Torrejon , M. Riou , F. A. Araujo , S. Tsunegi , G. Khalsa , D. Querlioz , P. Bortolotti , V. Cros , K. Yakushiji , A. Fukushima , H. Kubota , S. Yuasa , M. D. Stiles , J. Grollier , Nature 2017, 547, 428. 28748930 10.1038/nature23011 PMC5575904

[43] J. Dambre , D. Verstraeten , B. Schrauwen , S. Massar , Sci. Rep. 2012, 2, 1. 10.1038/srep00514 PMC3400147 22816038

[44] T. Kubota , H. Takahashi , K. Nakajima , Phys. Rev. Res. 2021, 3, 043135.

[45] H. Jaeger , Short term memory in echo state networks, GMD‐Forschungszentrum Informationstechnik, Sankt Augustin, 2001, p. 5.

[46] H. Jaeger , Bonn, Germany: German National Research Center for Information Technology GMD Technical Report 2001, 148, 13.

[47] K. Xing , Y. Wang , Q. Zhu , H. Zhou , Control Eng. Pract. 2012, 20, 477.

[48] Y. Cao , J. Huang , IEEE/CAA J. Autom. Sin. 2020, 7, 1478.

[49] W. Sun , N. Akashi , Y. Kuniyoshi , K. Nakajima , in The 32nd 2021 International Symposium on Micro‐NanoMechatronics and Human Science, IEEE, Piscataway, NJ 2021, pp. 1–6.

[50] W. Sun , N. Akashi , Y. Kuniyoshi , K. Nakajima , in 2022 IEEE 5th International Conference on Soft Rob. (RoboSoft), IEEE, Piscataway, NJ 2022, pp. 409–415.

[51] W. Sun , N. Akashi , Y. Kuniyoshi , K. Nakajima , IEEE Robotics and Autom. Lett. 2022, 7, 6862.

[52] S. Hochreiter , J. Schmidhuber , Neural Comput. 1997, 9, 1735. 9377276 10.1162/neco.1997.9.8.1735

[53] A. J. Ijspeert , Neural Net. 2008, 21, 642. 10.1016/j.neunet.2008.03.014 18555958

[54] K. Nakajima , K. Fujii , M. Negoro , K. Mitarai , M. Kitagawa , Phys. Rev. Appl. 2019, 11, 034021.

[55] O. E. Rössler , Phys. Lett. A 1976, 57, 397.

[56] H. Hauser , A. J. Ijspeert , R. M. Füchslin , R. Pfeifer , W. Maass , Biol. Cybern. 2012, 106, 595. 22956025 10.1007/s00422-012-0516-4

[57] T.‐Y. Li , J. A. Yorke , Period Three Implies Chaos, Springer New York, New York 2004, pp. 77–84.

[58] K. Inoue , K. Nakajima , Y. Kuniyoshi , Sci. Adv. 2020, 6, abb3989. 10.1126/sciadv.abb3989 PMC7673744 33177080

[59] L. Larger , M. C. Soriano , D. Brunner , L. Appeltant , J. M. Gutiérrez , L. Pesquera , C. R. Mirasso , I. Fischer , Opt. Express 2012, 20, 3241. 22330562 10.1364/OE.20.003241

[60] N. Akashi , Y. Kuniyoshi , S. Tsunegi , T. Taniguchi , M. Nishida , R. Sakurai , Y. Wakao , K. Kawashima , K. Nakajima , Adv. Intell. Syst. 2022, 4, 2200123.

[61] K. Goto , K. Nakajima , H. Notsu , New J. Phys. 2021, 23, 063051.

[62] M. Ushio , K. Watanabe , Y. Fukuda , Y. Tokudome , K. Nakajima , R. Soc. Open Sci. 2023, 10, 221614. 37090968 10.1098/rsos.221614 PMC10113807

[63] H. Cai , Z. Ao , C. Tian , Z. Wu , H. Liu , J. Tchieu , M. Gu , K. Mackie , F. Guo , Nat. Electron. 2023, 6, 1032.

[64] D. P. Kingma , J. Ba , *arXiv*, 2017.

[65] C.‐P. Chou , B. Hannaford , IEEE Trans. Rob. Autom. 1996, 12, 90.

[66] B. Tondu , J. Intell. Mater. Syst. Struct. 2012, 23, 225.

[67] H. Chaoui , P. Sicard , W. Gueaieb , IEEE Trans. Ind. Electron. 2009, 56, 3174.

[68] W. Huang , X. Huang , C. Majidi , M. K. Jawed , Nat. Commun. 2020, 11, 1. 32376823 10.1038/s41467-020-15651-9 PMC7203284

[69] J. Y. Loo , Z. Y. Ding , V. M. Baskaran , S. G. Nurzaman , C. P. Tan , Soft Rob. 2021, 9, 591. 10.1089/soro.2020.0024 34171965

