# Supplementary material for: Embedding Bifurcations into Pneumatic Artificial Muscle
Source: Adv Sci (Weinh). 2024 Apr 19;11(25):2304402. doi: 10.1002/advs.202304402 (PMC11220718; doi:10.1002/advs.202304402)
Supplement: Supplementary file 1 — Supporting Information [file ADVS-11-2304402-s002.pdf]

ADVANCED SCIENCE
Open Access

Supporting Information

for *Adv. Sci.*, DOI 10.1002/advs.202304402

Embedding Bifurcations into Pneumatic Artificial Muscle

Nozomi Akashi, Yasuo Kuniyoshi, Taketomo Jo, Mitsuhiro Nishida, Ryo Sakurai, Yasumichi Wakao and Kohei Nakajima*

Supporting Information: Embedding bifurcations into pneumatic artificial muscle

Nozomi Akashi Yasuo Kuniyoshi Taketomo Jo Mitsuhiro Nishida Ryo Sakurai Yasumichi Wakao Kohei Nakajima*

N. A.
Graduate School of Informatics, Kyoto University, Yoshida-honmachi, Sakyo-ku, 606-8501, Kyoto, Japan
Email Address: akashi.nozomi.2a@kyoto-u.ac.jp
Y. K., K. N.
Graduate School of Information Science and Technology, The University of Tokyo, 7-3-1 Hongo, Bunkyo-ku, 113-8654, Tokyo, Japan
T. J., M. N., R. S., Y. W.
GX Innovation Technology Development, Bridgestone Corporation, 3-1-1 Kyobashi, Chuo-ku, 104-8340, Tokyo, Japan

1 Dependencies of information-processing capacities on controller, body, and environmental conditions

Due to the concept of embodiment, the information-processing capability of a pneumatic artificial muscle (PAM) cannot be evaluated based only on the controller; thus, it is necessary to consider the interactions between the controller, body, and environment [1]. The dependency of the information processing capacity (IPC) on external load conditions in the main text has been focused on the relationship between the information processing ability of the system and the environment. In this section, we analyze the dependencies of the IPC on the i) input interval; ii) input magnitude; iii) number of time-multiplexing, which are the parameters in the controller, iv) equilibrium length of the PAM, which is a parameter in the body; and v) temperature, which is a parameter in the environment. The base condition of the experiments is presented in Table S1 below, and the parameters that are not in focus are fixed to the value in the table for all experiments.

Table S1: Base conditions of the experiment

Controller			Body	Environment	
Input interval	Input magnitude	The number of time-multiplexing	Equilibrium length	Load	Temperature
0.1 sec	0.5 MPa	5	108 mm	100 N	20 °C

1.1 Input interval

We analyzed the dependency of the IPC in the input interval. In addition, we analyzed the five conditions of $\tau = 0.04, 0.10, 0.16, 0.22$, and 0.28 sec as the input intervals. The results are depicted in Fig. S1. The delay components drastically changed through the changing of the input interval. When the input interval is 0.04 sec, delay components 1 and 2 were dominant. However, when the input interval was larger than 0.16 sec, almost all delay components were 0. It is evident that the current input could not sufficiently reflect when the input interval was small; on the other hand, past inputs decayed when the input interval was large. In addition, the degree components changed slightly after a change in the input interval.

1.2 Input magnitude

Next, we confirmed the dependency of the IPC on the input magnitude. We analyzed the five conditions, $A = 0.1, 0.2, 0.3, 0.4$, and 0.5 MPa, as the input magnitude. The results are shown in Fig. S1. The

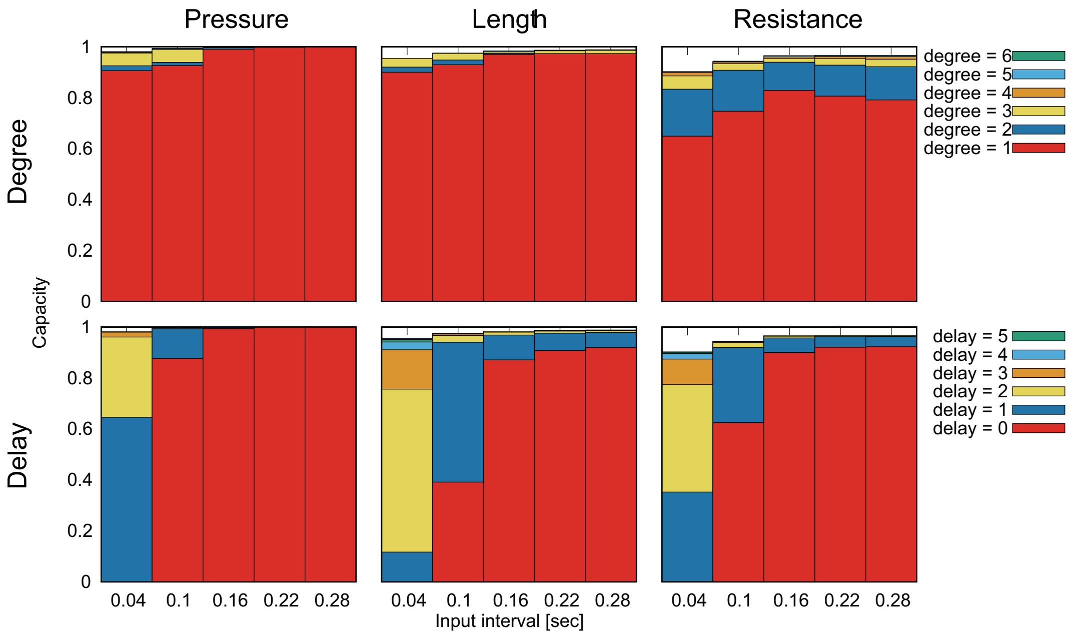

Figure S1: IPCs through the input interval. These are separated by degree (top graphs) and delay (bottom graphs) components.

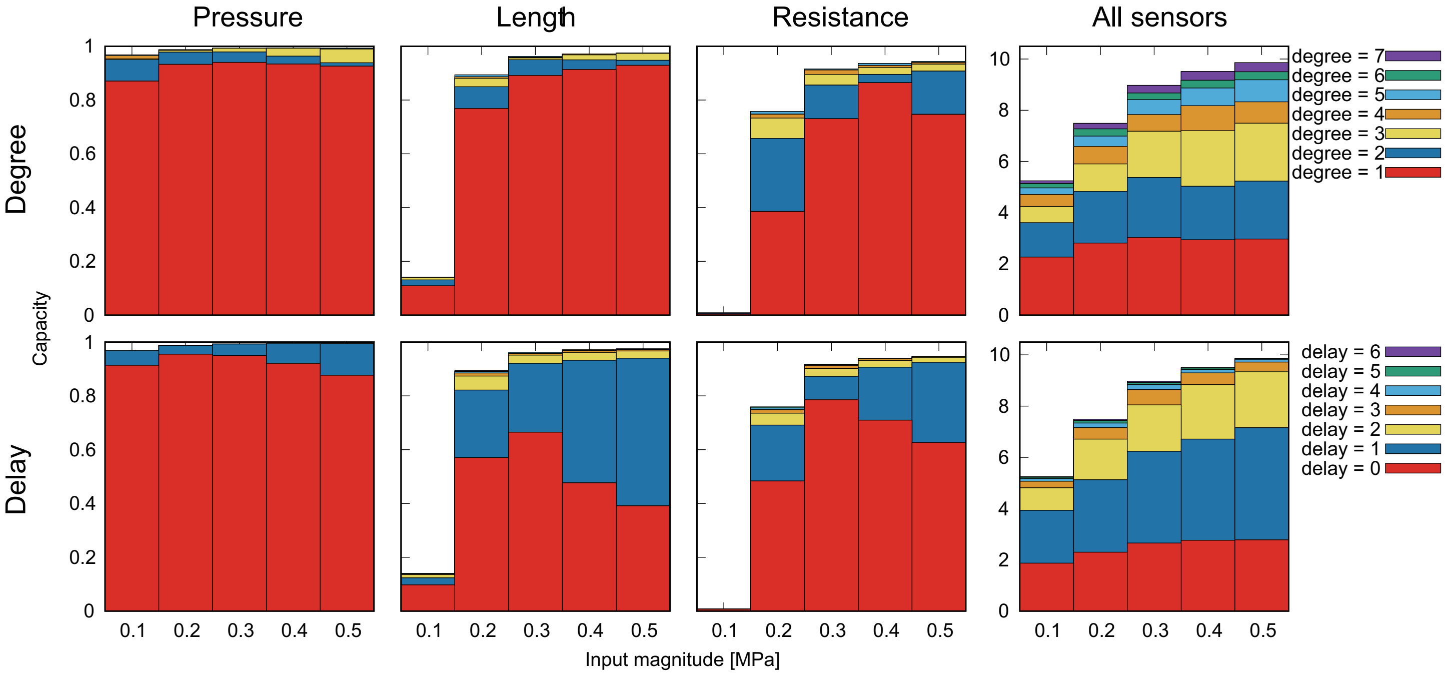

Figure S2: IPCs through the input magnitude. These are separated by degree (top graphs) and delay (bottom graphs) components.

IPCs of length and resistance were much lower than 1 when the input magnitude was 0.1 or 0.2 MPa. These results were caused by the deadband [2, 3, 4], thereby revealing that the dynamics of the PAM were barely responsive to the small input. When the input magnitude was larger than 0.3 MPa, the IPCs of length and resistance fill in 1. Therefore, it was suitable to actuate the PAM with a certain magnitude that was larger than 1 in the deadband. In addition, the IPC of all sensors combined slowly increasesed as the input magnitude increasesed.

1.3 The number of time-multiplexing

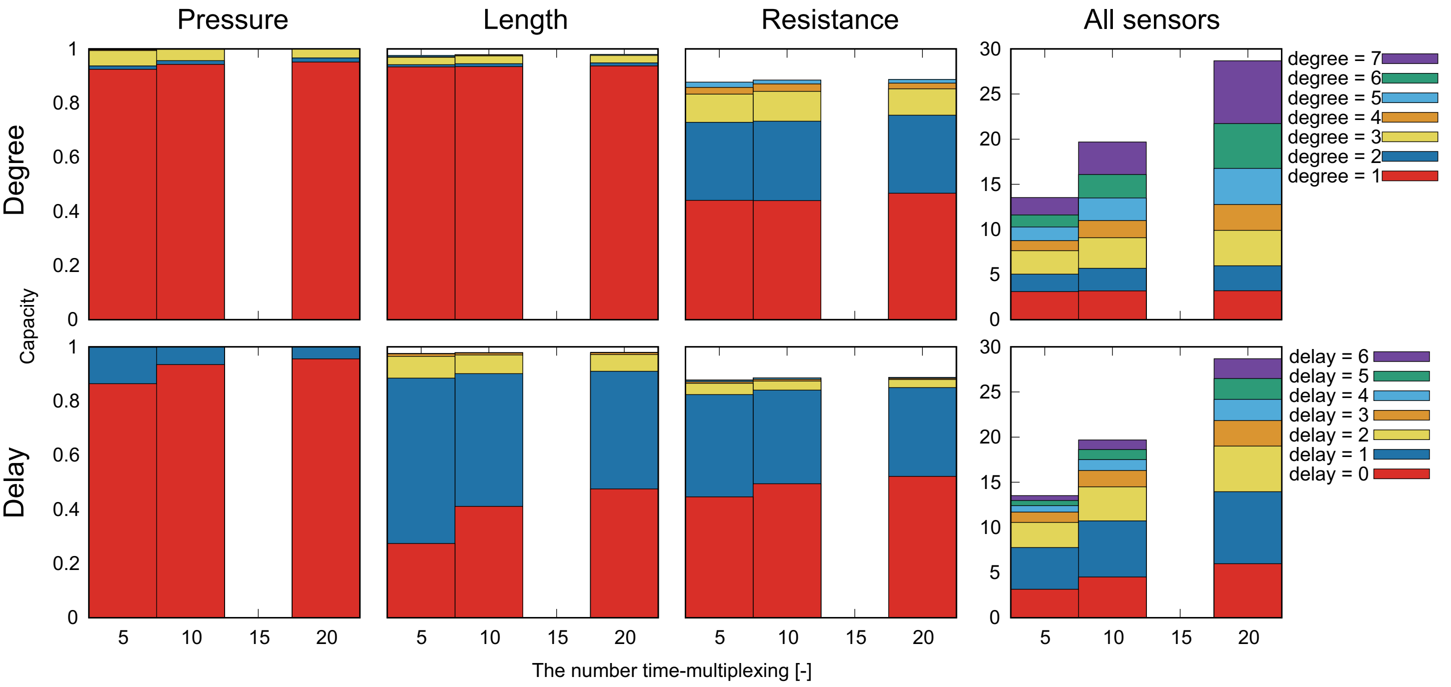

Figure S3: IPCs through the number of time-multiplexing. These are separated by degree (top graphs) and delay (bottom graphs) components.

Here, we confirmed the dependency of the IPC on the number of time-multiplexing. The time-multiplexing method was introduced in the main text. The number of time-multiplexing is represented as L in Eq. (4) in the main text. This number is restricted to the measurement frequency of sensory values. We used a digital multimeter for electric resistance measurement. In the analyses found in this subsection, we used the applying constant voltage source-based method as the electrical resistance measurement. Detailed information on these measurements is provided in the experimental section in the main text. We analyzed the three conditions: $L = 5, 10$, and 20 MPa. These conditions corresponded to the measurement frequencies 50 Hz, 100 Hz, and 200 Hz. The results are shown in Fig. S3. We confirmed that IPC in all sensors increases by increasing the number of time-multiplexing in the range from 5 to 20. Doubling the number of time-multiplexing will double the number of computational nodes, but will not double the IPC. This suggests that the rank of reservoir time series $\mathcal{X}$ does not fully increase through after an increase in the number of time-multiplexing.

1.4 Equilibrium length

In this subsection, we confirm the dependency of the IPC on the equilibrium length of the PAM. We analyzed the three conditions in which the equilibrium lengths of the PAM are $l_0 = 54, 108$, and 162 mm. The results are depicted in Fig. S4. The IPCs of all conditions were almost the same. The components of length and resistance changed at different equilibrium lengths, while the component of pressure did not. This is probably because the response time for the input pressure became longer, and the previous

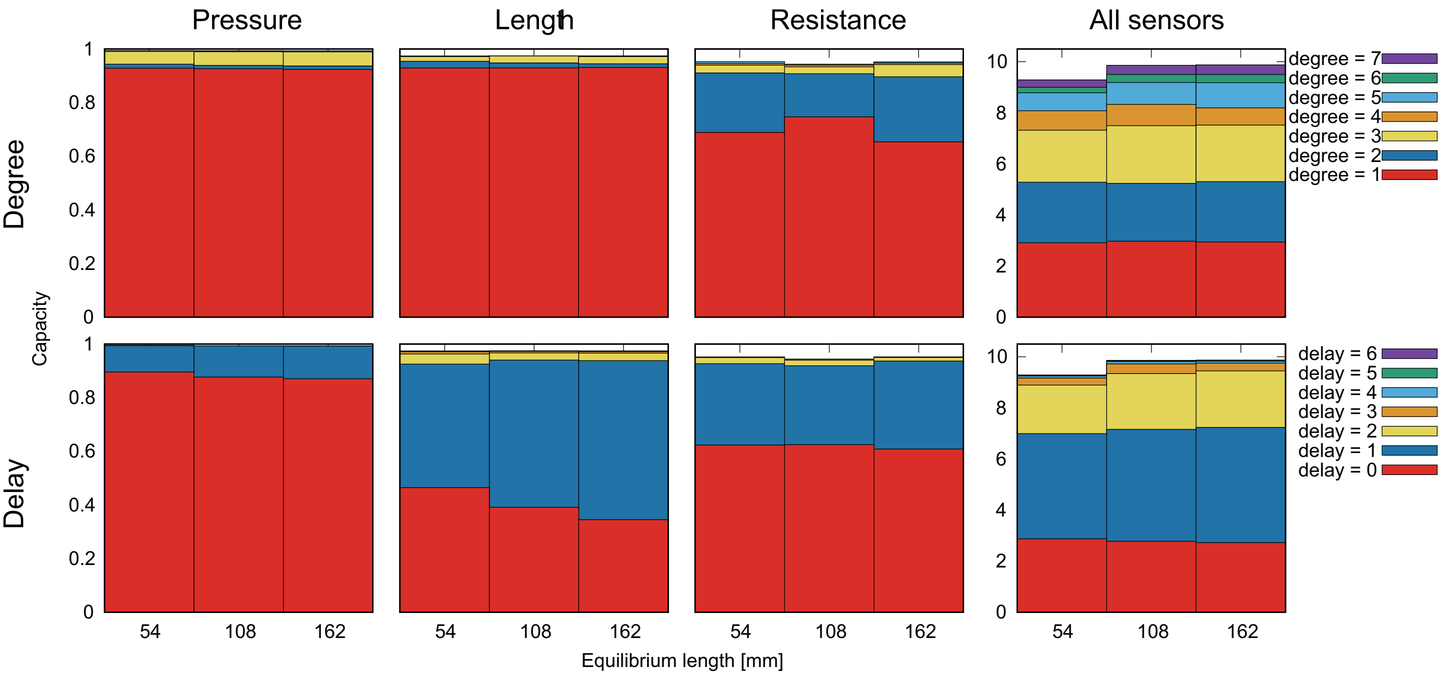

Figure S4: IPCs through the equilibrium length. These are separated by degree (top graphs) and delay (bottom graphs) components.

inputs remained much when the PAM became longer. On the other hand, the delay components of resistance did not change much at different equilibrium lengths, though the degree components of resistance changed slightly. The thickness of the PAM at different equilibrium lengths was different with the same input pressure, and the dynamics of the PAM could change because of the bifurcations of resistance, as mentioned in the main text.

1.5 Temperature

Finally, we analyzed the dependency of the IPC on temperature. We treat four temperature conditions: 0°C, 10°C, 23°C, and 40°C. The results are depicted in Fig. S5. The IPCs of all conditions were almost the same. However, the delay components of length and resistance changed based on the temperature. The elastic modulus of the rubber depended on the temperature and increased as the temperature increased [5]. Therefore, if the temperature was higher, the response of the input pressure was smaller and was the same as the response of a small input magnitude case.

2 Robustness against observational noise in the PAM length sensor emulation task

Here, we conducted an analysis of robustness against observational noise in the PAM length sensor emulation task discussed in Section 2.6 of the main text. We considered noise in data in the following form:

$$\mathbf{x}_n + \mathbf{p}_n, \tag{S1}$$

where $\{\mathbf{x}_n\}_n$ is the reservoir variable and $\{\mathbf{p}\}_n$ is the noise signal from the Gaussian distribution. We analyze the normalized mean squared error (NMSE) through the signal-noise (SN) ratio (A_s/A_n), where A_s and A_n are variances of the reservoir variable and noise, respectively. Fig. S6 shows the results of the analysis. PAM PRC was more directly affected by noise, leading to a decrease in performance compared to ESN. We can assume that ESN nodes behaved according to the same activation function, creating a somewhat redundant configuration that is robust to noise. On the other hand, PAM, with each physical

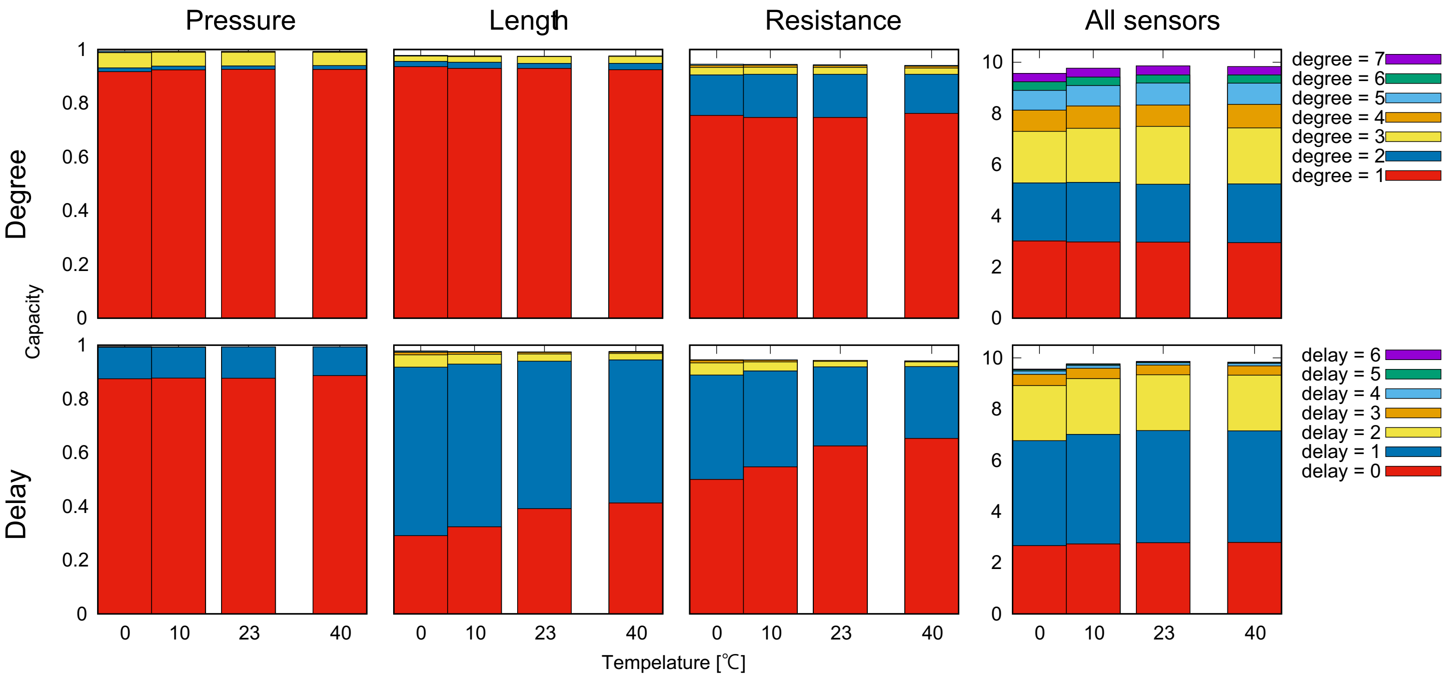

Figure S5: IPCs through temperature. These are separated by degree (top graphs) and delay (bottom graphs) components.

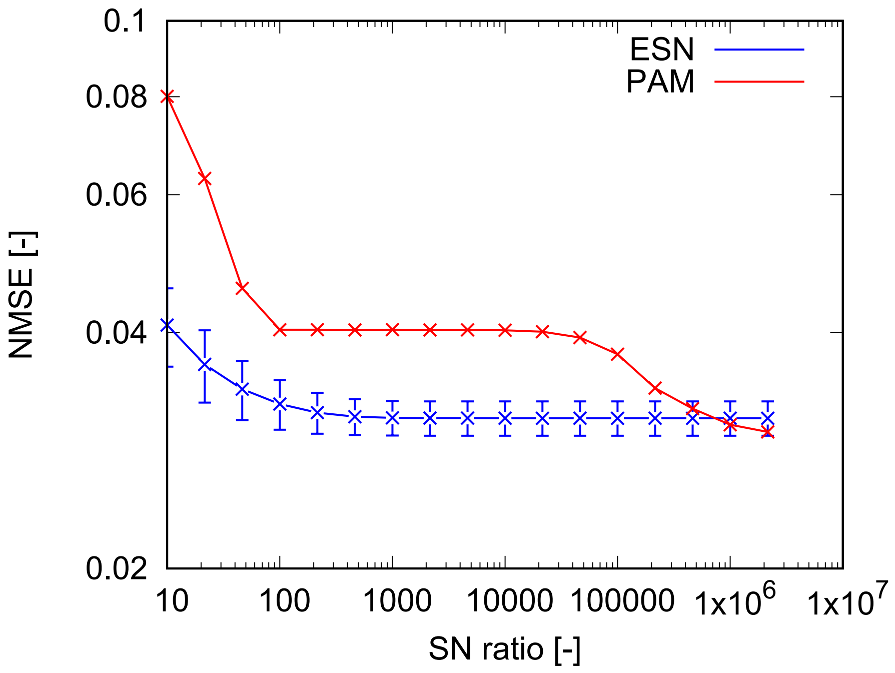

Figure S6: NMSEs of the PAM length sensor emulation task depending on the SN ratio.

variable exhibiting entirely different behavior, was susceptible to the critical effects of noise on specific variables.

3 Nonlinear autoregressive moving average task

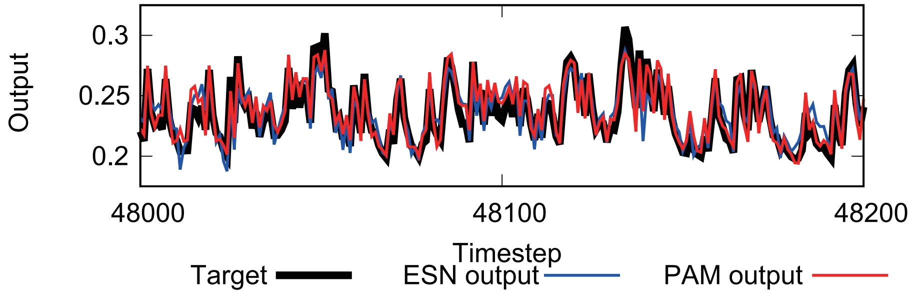

Figure S7: Time series of the target and output signals in the NARMA2 task. The number of ESN nodes is 20.

Here, we evaluated the performance of the PAM PRC in a typical benchmark task for recurrent neural networks. The task we treated is a second-order *nonlinear autoregressive moving average* (NARMA2) [6]. NARMA2 is an emulation task with the following difference equation:

$$y_{k+1} = \alpha y_k + \beta y_k y_{k-1} + \gamma z_k^3 + \delta, \quad \text{(S2)}$$

$$z_k = \mu + \sigma u_k, \quad \text{(S3)}$$

where $\{y_k\}_{k=1,\dots}$ is the target signal, $\{u_k\}_{k=0,\dots}$ is the input signal, and $(\alpha, \beta, \gamma, \delta, \mu, \sigma)$ are the parameters of NARMA2. We used the parameters $(\alpha, \beta, \gamma, \delta, \mu, \sigma) = (0.4, 0.4, 0.6, 0.1, 0.25, 0.25)$ [6, 7, 8]. NARMA2 requires nonlinear transformation and memory. In this case, the input sequence is comprised of uniformly random values and is transformed to [0, 0.5] MPa for the control pressure value.
Furthermore, we explain the reservoir variables in the PAM for each task. We used sensor values of pressure, length, resistance, and load as reservoir variables, and we multiplexed these sensor values by time multiplexing $L = 5$. Therefore, the number of reservoir variables was 20.

Table S2: NMSEs in the NARMA2 task

Task	System		
	ESN	PAM PRC without loads	PAM PRC with loads
NARMA2	0.1701	0.125	0.124

The NMSE of the task is presented in Table S2. The PAM PRC outperformed the ESN. We show the output and prediction time series of the second-order NARMA2 task in Fig. S7. The input time series of this task was the same as the length sensor emulation task in the main text. We can confirm that the prediction of the PAM physical reservoir computing was more consistent with the target than the ESN prediction.

4 Quantitative evaluation of logistic attractor embedding

This section presents quantitative analyses of attractor embedding in Fig. 6 in the main text and compares performances with ESNs. Attractor embedding via reservoir computing can be comprehensively understood by applying theories of *topologically conjugate* in dynamical systems [9, 10]. However, evaluating these theoretical concepts in experimental scenarios is challenging, and previous studies of attractor embedding have used multiple criteria to compare characteristic values of target and prediction dynamical systems [11, 12, 10]. Here, we calculate the following three criteria which are visually shown in Fig. 6A, 6B, and 6C.

- Valid time. The time when the target and prediction are continuously sufficiently close.
$$\lambda \max\{T \geq 0 \mid |y_{t_{\mathrm{sw}}+t} - \tilde{y}_{t_{\mathrm{sw}}+t}| < \varepsilon (0 \leq t \leq T)\}, \tag{S4}$$
where, λ is the largest Lyapunov exponent of the target system, and t_{sw} is the time step of switching from open loop to closed loop. We use the valid error threshold $\varepsilon = 0.05$ here.

- Attractor error. Distance between the target attractor and prediction signal in delay coordinate.
$$\frac{1}{T_{\mathrm{eval}}} \sum_{t=t_{\mathrm{sw}}+1}^{t_{\mathrm{sw}}+T_{\mathrm{eval}}} ||f(\tilde{y}(t-1)) - \tilde{y}(t)|| \tag{S5}$$
where T_{eval} is the evaluation timestep, and f is the map of the target system.

- Fourier spectral error. Fourier spectral distance between the target and prediction signal.
$$\frac{1}{M} \sum_{m=1}^{M} |g(s_m) - \tilde{g}(s_m)| \tag{S6}$$
where M is the number of spectra made from signals by fast Fourier transformation, and g and $\tilde{g}$ are Fourier spectra of target and prediction signals, respectively.

The focus here is on the logistic attractor as the target, and we compare the performance with ESNs The computational nodes in PAM PRC, whose number is 100, are made by time-multiplexing and are not fully linear-independent. In addition, PAM PRC is exposed to various noises such as observational, controlling, and physical noises. For a fair comparison, we vary the number of nodes in ESNs and introduce observational noise to ESNs following the same procedure as outlined in Eq. S1. The results are presented in Table S3 and Fig. S8. PAM PRC demonstrates comparable performance with ESNs when $N = 20$ and when ESNs include noise with an SN ratio of 31.6 for these aspects. When $N < 20$ or SN ratio $<$ 31.6 for ESNs, the embedding of chaotic dynamics and attractor and Fourier spectrum errors exhibit significantly high values. Consequently, even without any pattern generators, the PAM provides the above computational capability for chaotic pattern generation.

Table S3: Performances of logistic attractor embedding

	Criterion		
System	Valid Time [-]	Attractor error [-]	Fourier spectral error [-]
PAM PRC	2.11	0.0617	0.00162
ESN $N = 100$ without noise	4.25	0.0550	0.00157
ESN $N = 20$ without noise	3.16	0.0551	0.00163
ESN $N = 100$ with noise SN ratio 31.6	1.72	0.0643	0.00176

5 Embedding complicated attractors into PAMs

In this section, we analyze embedding further complicated attractors into the PAM. The target attractors were the Van der Pol oscillator and the Rössler attractor [13]. The Van der Pol oscillator showed a limit cycle and is expressed as the following equation:
$$\begin{aligned} \dot{y}^1 &= y^2 \\ \dot{y}^2 &= -y^1 + (1-(y^1)^2)y^2. \end{aligned} \tag{S7}$$
The Rössler attractor of three-dimensional chaos with continuous time is defined by the following equation:
$$\begin{aligned} \dot{y}^1 &= -y^2 - y^3 \\ \dot{y}^2 &= y^1 + ay^2 \\ \dot{y}^3 &= b + y^1 y^3 - cy^3, \end{aligned} \tag{S8}$$

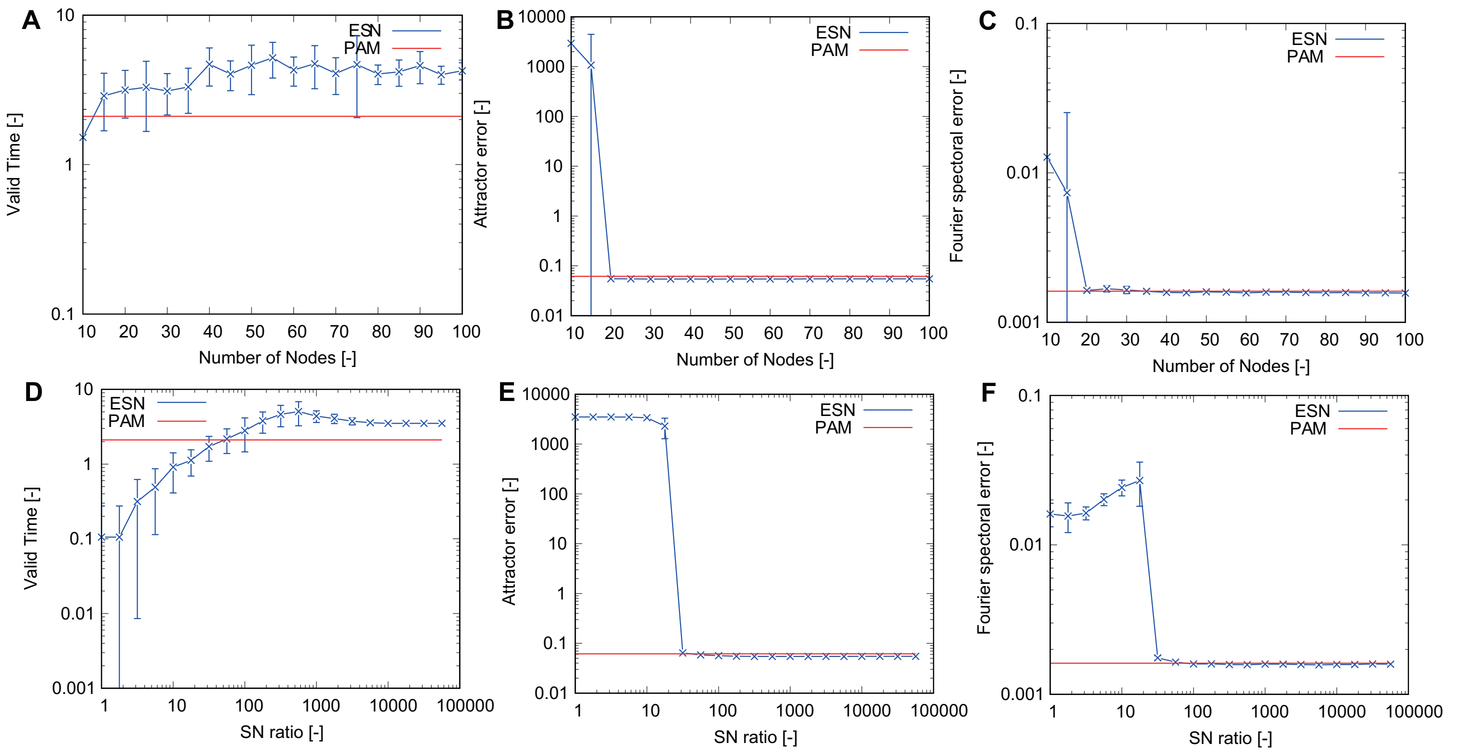

Figure S8: Comparisons of closed-loop logistic attractor embedding between ESNs and PAM PRC. A, B, and C are valid time, attractor error, and Fourier spectral error through the number of ESN nodes, respectively. D, E, and F are valid time, attractor error, and Fourier spectral error through SN ratio of observational noises in ESNs.

where a, b, and c are model parameters and set as typical chaotic parameters: $(a, b, c) = (0.2, 0.2, 5.7)$. Because chaos with continuous time does not occur unless it is a dynamical system with at least three dimensions [14] and the nonlinear term, which is essential for chaos, is only $y_1 y_3$, this model can be considered one of the simplest in chaos with continuous time.

The settings of the training and prediction phases were basically the same as the experiments of embedding Hénon attractor, which used the spatial multiplexing method. We used y^1 as an input signal of the Van der Pol and Rössler systems, which are multi-dimensional systems, and discretized the input signal for the Van der Pol and Rössler systems by a sampling interval of 0.4 and 0.5, respectively. Although the input was one-dimensional, the attractor can be reconstructed with all dimensions because of Takens' embedding theorem [15] if the reservoir has sufficient memory and nonlinearity. The input intervals of the PAM control pressure for the Van der Pol and Rössler systems, embedding were $\tau = 0.2$ and 0.3 sec, respectively.

Fig. S9 and S10 present the results of the attractor embedding. In the Van der Pol experiment, the attractor was almost entirely reconstructed, and the Fourier spectrum also exhibited matching peak components. In the open-loop setting of the Rössler attractor, PAM PRC was able to predict the target signals quite well. When transitioning to the closed-loop setting, the oscillatory dynamics of y^1 and y^2 were effectively reproduced, but the pulse dynamics of y^3 did not reconstruct as effectively. While the reconstructed attractor did not perfectly match the original one, it was still capable of capturing the complex structure of the Fourier spectrum, indicating partial reconstruction of the target high-dimensional chaotic structure.

6 Output weights analysis for closed-loop bifurcation embedding

Here, we analyze the relationship between the intrinsic bifurcations of reservoir dynamics and the bifurcation embedded in closed-loop reservoirs. We observed that the PAM had resistance bifurcations through changes in external loads, resulting in dramatic changes in the information processing capabil-

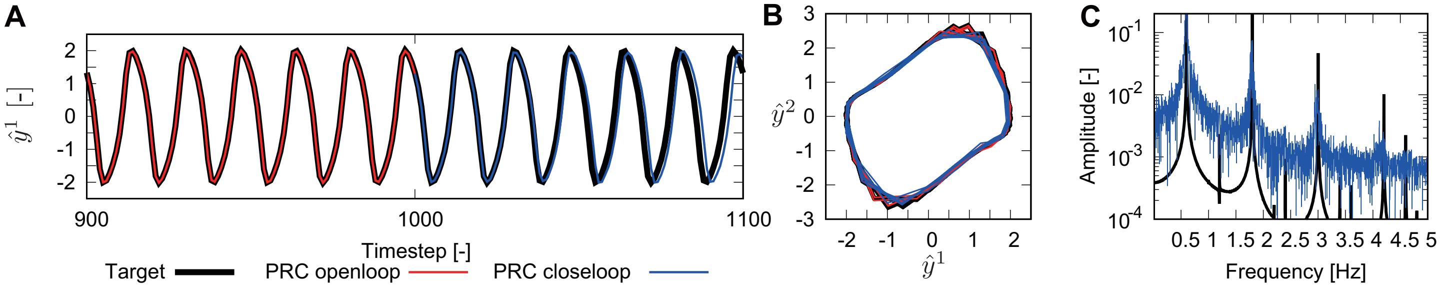

Figure S9: Results of the closed-loop at the Van der Pol oscillator embedding. A. Time series of the target and PRC output signals. B. Attractors of the target and PRC output. C. Spectra of the target and PRC output.

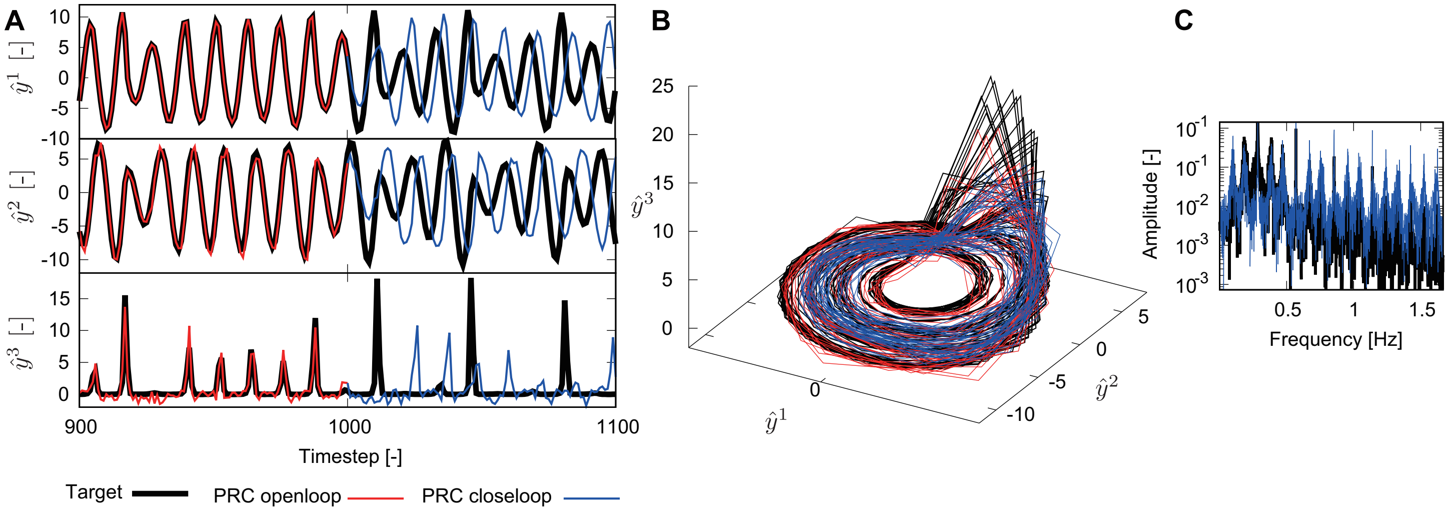

Figure S10: Results of the closed-loop at the Rössler attractor embedding. A. Time series of the target and PRC output signals. B. Attractors of the target and PRC output. C. Spectra of the target and PRC output.

ities of resistance. We aim to investigate how this resistance bifurcation contributes to the embedded bifurcation through the analysis of the output weights W_{out}.

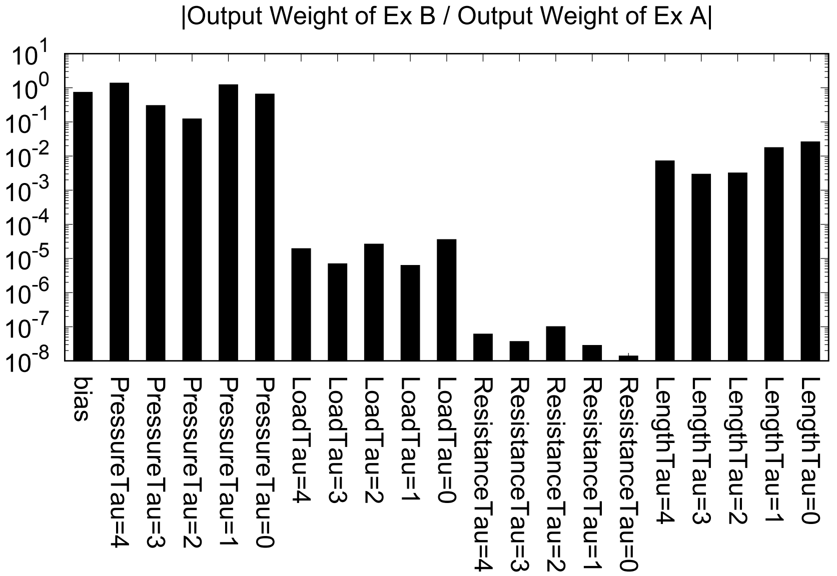

Figure S11: The ratios of output weight elements $|w_i^B/w_i^A|$ in experiments A and B in Section 2.8.

In Section 2.8 in the main text, we obtained results from experiment A, which induced bifurcation embedding when embedding a limit cycle, and experiment B, which did not induce embedded bifurcation. Fig. S8 shows the ratios of each component $|w_i^B/w_i^A|$ of the output weights $W_{\text{out}}^A = w_i^A$ from experiment A and $W_{\text{out}}^B = w_i^B$ from experiment B. As the figure illustrates, in experiment B, the output weights corresponding to resistance values were more than 10^{-7} times smaller compared to experiment A. Therefore, it is evident that the dramatic change in information processing capabilities of resistance values through the load in Fig. 3 contributed to the closed-loop bifurcation. Conversely, not actively using resistance values as reservoir variables allowed the suppression of bifurcation, as observed in experiment B.

7 Embedding periodic and chaotic attractors into the same weight

In this section, we demonstrate that we can embed two qualitatively different target signals into a single PAM through a change in the external load. In the section on the bifurcation embedding in the main text, we have shown that certain bifurcation structures can be embedded into a single PAM through the external load as a bifurcation parameter. These experiments did not show embedding the desired bifurcation structure but instead a bifurcation structure based on training data and reservoir dynamics. Here, we show that we can embed a bifurcation structure, which includes the desired target signal, by explicitly training qualitatively different target signals.

Fig. S12 presents the experimental results. In this experiment, we trained the chaotic and periodic dynamics of the logistic map with $a = 3.7$ and 3.2, which is described in Eq. (9) in the main text, when the external load was 100 N and 250 N, respectively. We controlled the single PAM by closed-loop using the trained output weight and observed its dynamics when the external load was switched from 100N to 250N at the 3,000th time step. The black and blue lines in S12A, B, and C before the 3,000th time step represent the time series, trajectories in delay coordinates, and frequency spectra, respectively, of the target signal and the PAM PRC output when the external load was 100 N. Moreover, the target signal and PAM PRC output have the same properties as chaos. The green lines in S12A, B, and C after the

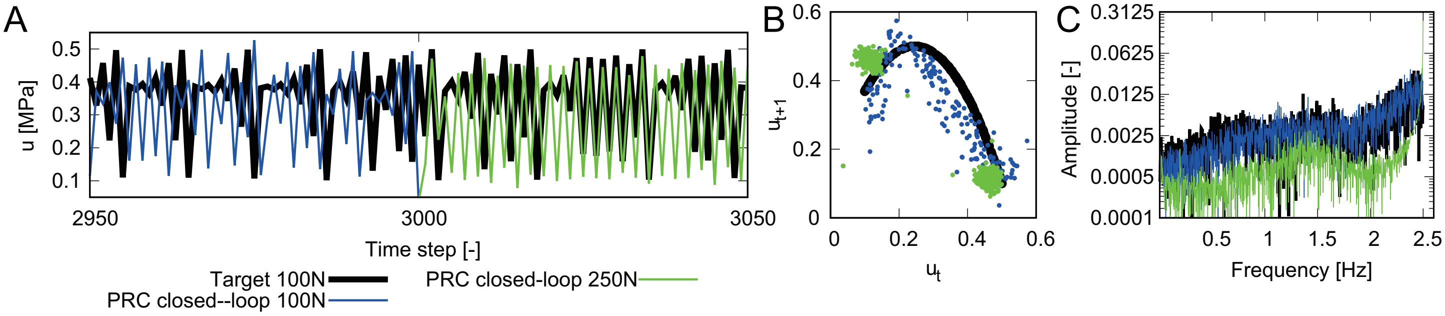

Figure S12: The results of the closed-loop at the chaos and period dynamics embedding. A. Time series of the target and PRC output signals. B. Attractors of the target and PRC output. C. Spectra of the target and PRC output.

3,000th time step show the time series, trajectories in delay coordinate, and frequency spectra, respectively, of the target signal and the PAM PRC output when the external load is 250 N. The PAM PRC output was quickly switched to period 2 dynamics, which is trained in 250 N, after the external load was switched to 250 N. Therefore, we can embed a bifurcation structure that includes the desired dynamics by explicitly training the dynamics on both sides of bifurcation.

References

[1] R. Pfeifer, C. Scheier, *Understanding intelligence*, MIT press, Cambridge, **2001**.

[2] T. V. Minh, T. Tjahjowidodo, H. Ramon, H. Van Brussel, *Mechatronics* **2010**, *20*, 3 402.

[3] M. Hofer, R. D'Andrea, In *2018 IEEE/RSJ International Conference on Intelligent Robots and Systems (IROS)*. **2018** 1456–1463.

[4] W. Huang, X. Huang, C. Majidi, M. K. Jawed, *Nature communications* **2020**, *11*, 1 1.

[5] K. H. Meyer, C. Ferri, *Rubber Chemistry and Technology* **1935**, *8*, 3 319.

[6] A. F. Atiya, A. G. Parlos, *IEEE transactions on neural networks* **2000**, *11*, 3 697.

[7] H. Kubota, K. Yakushiji, A. Fukushima, S. Tamaru, M. Konoto, T. Nozaki, S. Ishibashi, T. Saruya, S. Yuasa, T. Taniguchi, et al., *Applied Physics Express* **2013**, *6*, 10 103003.

[8] K. Nakajima, H. Hauser, R. Kang, E. Guglielmino, D. G. Caldwell, R. Pfeifer, *Frontiers in computational neuroscience* **2013**, *7* 91.

[9] A. Hart, J. Hook, J. Dawes, *Neural Networks* **2020**, *128* 234.

[10] M. Hara, H. Kokubu, *Journal of Dynamics and Differential Equations* **2022**, 1–26.

[11] Z. Lu, B. R. Hunt, E. Ott, *Chaos: An Interdisciplinary Journal of Nonlinear Science* **2018**, *28*, 6 061104.

[12] J. Pathak, A. Wikner, R. Fussell, S. Chandra, B. R. Hunt, M. Girvan, E. Ott, *Chaos: An Interdisciplinary Journal of Nonlinear Science* **2018**, *28*, 4 041101.

[13] O. E. Rössler, *Physics Letters A* **1976**, *57*, 5 397.

[14] I. Bendixson, *Acta Mathematica* **1901**, *24*, 1 1.

[15] F. Takens, In *Dynamical systems and turbulence, Warwick 1980*, 366–381. Springer, **1981**.
